# Supplementary material for: Separation of scales and a thermodynamic description of feature learning in some CNNs
Source: Nat Commun. 2023 Feb 17;14:908. doi: 10.1038/s41467-023-36361-y (PMC9938275; doi:10.1038/s41467-023-36361-y)
Supplement: Supplementary file 1 — Supplementary Information [file 41467_2023_36361_MOESM1_ESM.pdf]

Supplementary Information - Separation of Scales and a Thermodynamic Description of Feature Learning in Some CNNs

Contents

1 Supplementary Note - Derivation of the Mean-field Equations for a Fully Connected Network 2

1.1 Model Definition and Main Players . . . 2
1.2 Mean-Field Decoupling . . . 5
1.2.1 Mean-field Decoupling - Hidden Layers . . . 5
1.2.2 Mean-Field Decoupling - Output Layer . . . 6
1.3 Variational Gaussian approximation . . . 8
1.3.1 $l \in [2, L-1]$ . . . 9
1.3.2 $l = 1$ . . . 9
1.4 Equations of State (EoS) . . . 10
1.4.1 Extension to test points . . . 11
1.4.2 Auxiliary field correlation $\langle \mathbf{m}^{(l)}(\mathbf{m}^{(l)})^{\mathsf{T}} \rangle_{\text{MF}}$ . . . 13
1.5 An Emergent Scale . . . 14
1.6 Estimating Corrections to Mean-field Results . . . 16

2 Supplementary Note - Mean-Field Equations for CNNs 18

2.1 An Emergent Scale in CNNs . . . 22
2.2 Estimating Mean-field corrections - CNNs . . . 23

3 Supplementary Note - Toy Example - One Hidden Layer 24

3.1 Continuum Limit of Summations for CNNs and FCNs . . . 29

4 Supplementary Note - Validity of the Variational Gaussian Approximation 31

5 Supplementary Note - Variational Gaussian Approximation for ReLU Activation 34

6 Supplementary Note - Further details on the numerical experiments 35

6.1 FCN experiments . . . 35
6.2 2-layer CNN experiment . . . 36
6.3 Myrtle-5 CNN on subsets of CIFAR-10 experiment . . . 38

1 Supplementary Note - Derivation of the Mean-field Equations for a Fully Connected Network

In this section, we derive the equations of state for deep fully connected NNs with a finite number of layers, L. In Sec. 2 we provide a sketch of the derivation for CNN architecture. The analysis can also be extended to other architectures, including pooling layers and skip connections.

1.1 Model Definition and Main Players

The model is composed of a L layer NN having $L-1$ activated layers, and one linear readout layer. Specifically, we consider

$$f(\mathbf{x}) = \sum_{j=1}^{N_{L-1}} w_j^{(L)} \phi\left(h_j^{(L-1)}(\mathbf{x})\right) \tag{1}$$

$$h_j^{(l+1)}(\mathbf{x}) = \sum_{i=1}^{N_l} W_{ji}^{(l+1)} \phi\left(h_i^{(l)}(\mathbf{x})\right) \tag{2}$$

$$h_i^{(1)}(\mathbf{x}) = \sum_{j=1}^{d} W_{ij}^{(1)} x_j \tag{3}$$

where $l \in [1, L-1]$, the weights of the network are $\mathbf{w}^{(L)} \in \mathbb{R}^{N_{L-1}}$, $W^{(l)} \in \mathbb{R}^{N_{l-1}\times N_l}$, such that $N_0 = d$ and $N_L = 1$, and $\mathbf{x} \in \mathbb{R}^d$ is the input vector. Bold letters denote vector or tensor quantities. The function $\phi : \mathbb{R} \to \mathbb{R}$ is the activation function applied element-wise. In subsection 1.3, when deriving the equation of states, we take $\phi = \text{erf}$ for concreteness, other activation functions can also be considered. See section 5 for an extension to ReLU. The training data is denoted by $\mathcal{D}_n = \{\mathbf{x}_\mu, y_\mu\}_{\mu=0}^n$ and we use a square loss $\mathcal{L} = \sum_{\mu=1}^n (y_\mu - f(\mathbf{x}_\mu))^2$. The feature matrix, X_n, represents all the input samples, $\{\mathbf{x}_\mu\}_{\mu=1}^n$.

Our main object of interest is the following equilibrium distribution of the Langevin dynamics algorithm with noise strength σ^2 and weight decay written in function space (i.e. in terms of the DNNs outputs)

$$p(\mathbf{f}|\mathcal{D}_n) \propto p(\mathbf{f}|X_n)\text{exp}\left(-\frac{\mathcal{L}}{2\sigma^2}\right), \tag{4}$$

In practice, we sample from this distribution using Gradient descent (GD), at small learning rates, together with weight decay and noise on each weight derivative. The weight decay parameters are σ_l^2/N_{l-1} for layer l. The first term on the r.h.s. is given by

$$p(\mathbf{f}|X_n) = \left\langle \prod_\mu \delta\left[f(\mathbf{x}_\mu) - \sum_{j=1}^{N_{L-1}} w_j^{(L)} \phi\left(h_j^{(L-1)}(\mathbf{x}_\mu)\right)\right]\right\rangle_{\{W^{(l)}\}_{l=1}^{L-1}, \mathbf{w}^{(L)}} \tag{5}$$

where $\mathbf{f} = (f_1, ..., f_n)$ is viewed now as a random variable following the NN outputs on all different training points. The average $\langle ... \rangle_{\mathbf{w}}$ is over the weights $\mathbf{w}$ of the network at equilibrium. The weights' distribution at equilibrium can be obtained explicitly. At $N_l \to \infty$, for $l \in [1, L]$ these distributions (Eq. (5) and Eq. (4)) tend to a GP, however our interest here is at finite N_l.

Eq. (4) and Eq. (5) can also be understood from a Bayesian perspective. Eq. (4) can be viewed as the posterior distribution assuming each sample is generated by a neural network model as in Eq. (3) and is corrupted by an additive i.i.d. Gaussian noise with variance σ^2. The prior distribution over the weights of the network is taken to be Gaussian with variance σ_l^2/N_{l-1}.

To obtain a more explicitly "layer-wise" representation of Eq. 5, we next condition over the pre-activations ($\mathbf{h}^{(l)}$) of each layer using Bayes' formula, we obtain the following Markov representation of Eq. (5)

$$p(\mathbf{f}|X_n) = \int p(\mathbf{f}|\mathbf{h}^{(L-1)}, X_n) \prod_{l=1}^{L-2} p(\mathbf{h}^{(l+1)}|\mathbf{h}^{(l)}, X_n) d\mathbf{h}^{(l)} d\mathbf{h}^{(L-1)}. \tag{6}$$

The hidden layers probabilities above are defined as follows:

$$p(\mathbf{f}|\mathbf{h}^{(L-1)}, X_n) = \left\langle \prod_{\mu} \delta \left(f_\mu - \sum_{j=1}^{N_L} w_j^{(L)} \phi \left(h_{j\mu}^{(L-1)} \right) \right) \right\rangle_{\mathbf{w}^{(L)}}, \tag{7}$$

$$p(\mathbf{h}^{(l+1)}|\mathbf{h}^{(l)}, X_n) = \left\langle \prod_{\mu j} \delta \left(h_{j\mu}^{(l+1)} - \sum_{i=1}^{N_l} W_{ji}^{(l+1)} \phi \left(h_{i\mu}^{(l)} \right) \right) \right\rangle_{W^{(l+1)}}, \tag{8}$$

$$p(\mathbf{h}^{(1)}|X_n) = \left\langle \prod_{\mu i} \delta \left(h_{i\mu}^{(1)} - \sum_{j=1}^{d} W_{ij}^{(1)} x_{\mu,j} \right) \right\rangle_{W^{(1)}}, \tag{9}$$

where we write for short $h_{i\mu}^{(l)} = h_i^{(l)}(\mathbf{x}_\mu)$ for $l \in \{1, ..., L-1\}$, the latter being the random variables describing the argument of the activation function (pre-activation) of the lth layer at neuron i, on the $\mathbf{x}_\mu$ data-point. Later we will use this Markov structure of the distribution to decouple the different layers.

We continue our analysis by using the Fourier identity, which replaces the above delta functions by auxiliary fields, $\mathbf{t}, \{\mathbf{m}^{(l)}\}_{l=1}^{L-1}$, which are conjugated to $\mathbf{f}, \{\mathbf{h}^{(l)}\}_{l=1}^{L-1}$, respectively. To this end, the probability distribution over the network output given the input data can be written as follows.

$$p(\mathbf{f}|X_n) \propto \int d\mathbf{t} \prod_{l=1}^{L-1} d\mathbf{m}^{(l)} d\mathbf{h}^{(l)} \exp\Big(-\mathcal{S} \left(\mathbf{f}, \mathbf{t}, \{\mathbf{m}^{(l)}, \mathbf{h}^{(l)}\}_{l=1}^{L-1} \right) \Big) \tag{10}$$

where we adopt here physics notation, and define, $\mathcal{S} \left(\mathbf{f}, \mathbf{t}, \{\mathbf{m}^{(l)}, \mathbf{h}^{(l)}\}_{l=1}^{L-1} \right)$, as the action associated with this distribution. Collecting all terms, the action is

defined as follows

$$\mathcal{S} = \sum_{l=1}^{L-1} \mathcal{S}^{(l)} + \mathcal{S}_f \tag{11}$$

$$\mathcal{S}^{(1)} = \frac{1}{2}\sum_{\mu\nu i} m_{\mu i}^{(1)} m_{\nu i}^{(1)} Q_{\mu\nu}^{(1)} - i\sum_{\mu j} m_{\mu j}^{(1)} h_{\mu j}^{(1)}$$

$$\mathcal{S}^{(l)} = \frac{1}{2}\sum_{\mu\nu i} m_{\mu i}^{(l)} m_{\nu i}^{(l)} \tilde{Q}_{\mu\nu}^{(l)}(\mathbf{h}^{(l-1)}) - i\sum_{\mu j} m_{\mu j}^{(l)} h_{\mu j}^{(l)}$$

$$\mathcal{S}_f = -i\sum_{\mu} t_\mu f_\mu + \frac{1}{2}\sum_{\mu\nu} t_\mu t_\nu \left[\tilde{Q}_f(\mathbf{h}^{(L-1)})\right]_{\mu\nu} + \frac{1}{2\sigma^2}\sum_{\mu}(f_\mu - y_\mu)^2$$

To obtain the above expression, we performed Gaussian integration over the weights of all layers. In addition, we define the following matrices:

$$\tilde{Q}_f(\mathbf{h}^{(L-1)})_{\mu\nu} = \frac{\sigma_L^2}{N_{L-1}} \sum_{j=1}^{N_{L-1}} \phi(h_{j\mu}^{(L-1)})\phi(h_{j\nu}^{(L-1)}) \tag{12}$$

$$\tilde{Q}^{(l+1)}(\mathbf{h}^{(l)})_{\mu\nu} = \frac{\sigma_{l+1}^2}{N_l} \sum_{i=1}^{N_l} \phi(h_{i\mu}^{(l)})\phi(h_{i\nu}^{(l)}), \tag{13}$$

$$Q_{\mu\nu}^{(1)} = \frac{\sigma_1^2}{d}\mathbf{x}_\mu^\intercal \mathbf{x}_\nu. \tag{14}$$

Averages of these matrices within our mean-field theory defined below are referred to henceforth as post-kernels. The name post-kernel represents the fact that at large width, these matrices concentrate around the covariance matrix of the post activation. At infinite width, these are the GP kernels, but for finite width, we show that they concentrate around a different average, which is defined self-consistently via our mean-field theory. The action in Eq. (11) is associated with the partition function $\mathcal{Z} = \int e^{-\mathcal{S}} d\mathbf{f} d\mathbf{t} \Pi_{l=1}^{L-1} d\mathbf{h}^{(l)} d\mathbf{m}^{(l)}$. We comment that by integrating over the auxiliary fields, $\mathbf{t}, \mathbf{m}^{(l)}$, and using the following identity $\int e^{-\mathbf{x}^\intercal A\mathbf{x}/2+\mathbf{J}\mathbf{x}} d^n\mathbf{x} = \sqrt{\frac{(2\pi)^n}{\det A}} e^{\mathbf{J}^\intercal A^{-1}\mathbf{J}/2}$, where $\mathbf{J}, \mathbf{x} \in \mathbb{R}^n$, and $A \in \mathbb{R}^{n\times n}$, one obtains the equivalent form cited in the main text, containing only the pre-activations and the outputs

$$\mathcal{S} = \frac{1}{2}\sum_{\mu\nu i} h_{i\mu}^{(1)} \left[Q^{(1)}\right]_{\mu\nu}^{-1} h_{i\nu}^{(1)} + \frac{1}{2}\sum_{l=2}^{L-1}\sum_{\mu\nu i} h_{i\mu}^{(l)} \left[\tilde{Q}^{(l)}\right]_{\mu\nu}^{-1} h_{i\nu}^{(l)} + \frac{1}{2} f_\mu \left[\tilde{Q}_f(\mathbf{h}^{(L-1)})\right]_{\mu\nu}^{-1} f_\nu + \frac{1}{2\sigma^2}\sum_{\mu}(f_\mu - y_\mu)^2 \tag{15}$$

In the following section, we derive the inter-layer mean-field decoupling. In this context, the form of the action in the presence of the auxiliary fields (Eq. (11)) turns out to be useful.

1.2 Mean-Field Decoupling

Next, we capitalize on the fact that the coupling between layers in Eq. 11 is only through width-index averaged quantities, specifically the width-index average of $\mathbf{m}^{(l)}$ and $\tilde{Q}^{(l)}(\mathbf{h}^{(l-1)})$. Under such circumstances, it is natural to consider a mean-field type approximation where one replaces

$$\sum_{\mu\nu i} m_{\mu i}^{(l)} m_{\nu i}^{(l)} \tilde{Q}_{\mu\nu}^{(l)}(\mathbf{h}^{(l-1)}) \to \sum_{\mu\nu i} \langle m_{\mu i}^{(l)} m_{\nu i}^{(l)} \rangle_{\mathrm{MF}} \tilde{Q}_{\mu\nu}^{(l)}(\mathbf{h}^{(l-1)}) + \sum_{\mu\nu i} m_{\mu i}^{(l)} m_{\nu i}^{(l)} \langle \tilde{Q}_{\mu\nu}^{(l)}(\mathbf{h}^{(l-1)}) \rangle_{\mathrm{MF}} \tag{16}$$

where $\langle ... \rangle_{\mathrm{MF}}$ is the average over a mean-field distribution which we construct via the above approximation in a self-consistent manner. Importantly, following such a replacement, the fluctuations of the different layers as well as the different neurons within each layer become independent. This constitutes a major simplification and sets the stage for the final approximation we carry - a variational Gaussian approximation (VGA), to estimate the individual partition function of each neuron. This also allows us to derive the equation of states of the GP process we find. However, before turning to the VGA, the three subsections below provide a detailed derivation of the mean-field decoupling together with an estimate of the leading order correction to the mean-field action.

1.2.1 Mean-field Decoupling - Hidden Layers

Our first step is to understand the dependence of $\mathbf{h}^{(l)}$ on $\mathbf{h}^{(l-1)}$ for all $l \in [2, L-1]$. Following the mean-field idea introduced in Eq. (16) we first rewrite the action of the lth layer such that

$$\mathcal{S}^{(l)} = \frac{1}{2} \sum_{j\mu\nu} \langle m_{\mu j}^{(l)} m_{\nu j}^{(l)} \rangle_{\mathrm{MF}} \tilde{Q}_{\mu\nu}^{(l)}(\mathbf{h}^{(l-1)}) + \frac{1}{2} \sum_{\mu\nu j} m_{\mu j}^{(l)} m_{\nu j}^{(l)} \langle \tilde{Q}_{\mu\nu}^{(l)}(\mathbf{h}^{(l-1)}) \rangle_{\mathrm{MF}} + \frac{1}{2} i \sum_{\mu j} m_{\mu j}^{(l)} h_{\mu j}^{(l)} - \frac{1}{2} \sum_{\mu\nu} \Delta[\mathbf{m}^{(l)} (\mathbf{m}^{(l)})^{\mathsf{T}}]_{\mu\nu} \Delta \tilde{Q}_{\mu\nu}^{(l)}(\mathbf{h}^{(l-1)}) + \mathrm{const} \tag{17}$$

where $\Delta[\mathbf{m}^{(l)}(\mathbf{m}^{(l)})^{\mathsf{T}}]_{\mu\nu} = \frac{1}{N_l} \sum_j m_{\mu j}^{(l)} m_{\nu j}^{(l)} - \frac{1}{N_l} \sum_j \langle m_{\mu j}^{(l)} m_{\nu j}^{(l)} \rangle_{\mathrm{MF}}$, and $\Delta \tilde{Q}_{\mu\nu}^{(l)}(\mathbf{h}^{(l-1)}) = \tilde{Q}_{\mu\nu}^{(l)}(\mathbf{h}^{(l-1)}) - \langle \tilde{Q}_{\mu\nu}^{(l)}(\mathbf{h}^{(l-1)}) \rangle_{\mathrm{MF}}$. Note that in the mean-field decoupling $\frac{1}{N_l} \sum_j \langle m_{\mu j}^{(l)} m_{\nu j}^{(l)} \rangle_{\mathrm{MF}} = \langle m_{\mu j}^{(l)} m_{\nu j}^{(l)} \rangle_{\mathrm{MF}}$ for any $j \in [1, N_l]$, which due to the mean field decoupling this quantity no longer depend on the individual neuron j. The last term in Eq. (17) (discarding the *const*) is the fluctuations around the mean-field average. Since the sum over all neurons in the layer concentrates around its average for large width by the law of large numbers, the order of this term is $1/N_l$ smaller than all the other terms in the action. We, therefore, neglect this term. This leaves us with a Gaussian distribution over the auxiliary field, $\mathbf{m}^{(l)}$ which we can now

integrate over and obtain:

$$\int \prod_{j=0}^{N_l-1} d\mathbf{m}_j^{(l)} \exp\left(-\sum_{\mu\nu}\langle m_{\mu j}^{(l)} m_{\nu i}^{(l)}\rangle_{\text{MF}} \tilde{Q}_{\mu\nu}^{(l)}(\mathbf{h}^{(l-1)}) - \sum_{\mu\nu} m_{\mu j}^{(l)} m_{\nu i}^{(l)} \langle \tilde{Q}_{\mu\nu}^{(l)}(\mathbf{h}^{(l-1)})\rangle_{\text{MF}} - i\sum_{\mu} m_{\mu j}^{(l)} h_{\mu j}^{(l)}\right)$$
$$= \exp\left(-\frac{1}{2}\sum_{\mu\nu,j} h_{\mu j}^{(l)} [Q^{(l)}]_{\mu\nu}^{-1} h_{\nu j}^{(l)} - \frac{1}{2}\sum_{\mu\nu,j}\langle m_{\mu j}^{(l)} m_{\nu j}^{(l)}\rangle_{\text{MF}} \tilde{Q}_{\mu\nu}^{(l)}(\mathbf{h}^{(l-1)})\right). \tag{18}$$

In the second transition, we used the previously defined post-kernel $Q^{(l)} = \langle \tilde{Q}_{\mu\nu}^{(l)}(\mathbf{h}^{(l-1)})\rangle_{\text{MF}}$.

Following Eq. (18) for l and $l-1$, we gather all the $h^{(l-1)}$ dependent terms and obtain the mean-field action of the $l-1$th layer:

$$\mathcal{S}_{\text{MF}}^{(l-1)} = \frac{1}{2} N_l \sum_{\mu\nu} A_{\mu\nu}^{(l)} \tilde{Q}_{\mu\nu}^{(l)}(\mathbf{h}^{(l-1)}) + \sum_{\mu\nu j} \frac{1}{2} h_{\mu j}^{(l-1)} [Q^{(l-1)}]_{\mu\nu}^{-1} h_{\nu j}^{(l-1)} \tag{19}$$

where we denote by $A_{\mu\nu}^{(l)} = \langle m_{\mu j}^{(l)} m_{\nu j}^{(l)}\rangle_{\text{MF}}$, which due to the symmetry of the problem do not depend on the specific neuron j. The above mean-field action is valid for $l \in [2, L-1]$.

To calculate $A^{(l)} = \langle \mathbf{m}^{(l)} (\mathbf{m}^{(l)})^\top\rangle_{\text{MF}}$ for $l \in [2, L-1]$, we use standard multivariate Gaussian results (see subsection 1.4.2) this yields the following result:

$$A^{(l)} = [Q^{(l)}]^{-1}\left(I_n - \langle \mathbf{h}_j^{(l)} (\mathbf{h}_j^{(l)})^\top\rangle_{\text{MF}} [Q^{(l)}]^{-1}\right) \tag{20}$$

where $\langle \mathbf{h}_j^{(l)} (\mathbf{h}_j^{(l)})^\top\rangle_{\text{MF}}$ is determined self-consistently.

1.2.2 Mean-Field Decoupling - Output Layer

We next consider the coupling between the final/output layer and the penultimate layer. As done previously in the analysis of the hidden layers, we rewrite the action of the top layer as

$$\mathcal{S}_f = -i\sum_{\mu} t_\mu f_\mu + \frac{1}{2\sigma^2}\sum_{\mu}(f_\mu - y_\mu)^2 + \frac{1}{2}\sum_{\mu\nu}\langle t_\mu t_\nu\rangle_{\text{MF}}\left[\tilde{Q}_f(\mathbf{h}^{(L-1)})\right]_{\mu\nu}$$
$$+ \frac{1}{2}\sum_{\mu\nu} t_\mu t_\nu \left[\langle \tilde{Q}_f(\mathbf{h}^{(L-1)})\rangle_{\text{MF}}\right]_{\mu\nu} + \frac{1}{2}\sum_{\mu\nu}\Delta[t_\mu t_\nu]\left[\Delta\tilde{Q}_f(\mathbf{h}^{(L-1)})\right]_{\mu\nu} + \text{const} \tag{21}$$

where we define $\Delta[t_\mu t_\nu] = t_\mu t_\nu - \langle t_\mu t_\nu\rangle_{\text{MF}}$ and $\Delta\tilde{Q}_f(\mathbf{h}^{(L-1)}) = \tilde{Q}_f(\mathbf{h}^{(L-1)}) - \langle \tilde{Q}_f(\mathbf{h}^{(L-1)})\rangle_{\text{MF}}$.Unlike the hidden layers, here there is no averaging of many neurons, and therefore a-priori it is not clear whether the fluctuating term is small. Interestingly, we show in subsection 1.6 that this term leads to $O(1/N_{L-1})$

corrections in observable quantities. Following this analysis, we focus on the remaining terms, we can now integrate over the auxiliary field $\mathbf{t}$ and obtain:

$$\int d\mathbf{t} \exp\left(\sum_{\mu} it_\mu f_\mu - \frac{1}{2}\sum_{\mu\nu}\langle t_\mu t_\nu\rangle_{\text{MF}}\left[\tilde{Q}_f(\mathbf{h}^{(L-1)})\right]_{\mu\nu} - \frac{1}{2}\sum_{\mu\nu} t_\mu t_\nu \left[\langle\tilde{Q}_f(\mathbf{h}^{(L-1)})\rangle_{\text{MF}}\right]_{\mu\nu}\right)$$

$$= \exp\left(-\frac{1}{2}\sum_{\mu\nu} f_\mu [Q_f]^{-1}_{\mu\nu} f_\nu - \frac{1}{2}\sum_{\mu\nu}\langle t_\mu t_\nu\rangle_{\text{MF}}[\tilde{Q}_f(\mathbf{h}^{(L-1)})]_{\mu\nu} + \text{const}\right), \quad (22)$$

where in the second transition we define the output post-kernel as $Q_f = \langle\tilde{Q}_f(\mathbf{h}^{(L-1)})\rangle_{\text{MF}}$.

We stress that even in this regime, feature learning effects can still be of order, 1 as these are controlled by an emergent scale (χ) containing positive powers of n. See Fig. 1(c) in the main text.

For the $L-1$ layer, we obtain from Eq. (19) the following action:

$$\mathcal{S}_{\text{MF}}^{(L-1)} = \frac{1}{2}\sum_{\mu\nu} A^{(L)}_{\mu\nu}\tilde{Q}_f(\mathbf{h}^{(L-1)}) + \sum_{j\mu\nu}\frac{1}{2}h^{(L-1)}_{\mu j}[Q^{(L-1)}]^{-1}_{\mu\nu}h^{(L-1)}_{\nu j}, \quad (23)$$

where we denote by $A^{(L)}_{\mu\nu} = \langle t_\mu t_\nu\rangle_{\text{MF}}$. Notably, the fluctuations of the hidden layer fields (i.e. the $\mathbf{h}^{(L-1)}, \mathbf{m}^{(L-1)}$ variables) are now decoupled both from the input layer and the output layer. A tedious yet straightforward calculation similar to that carried in Sec. 1.4.2 reveals that

$$A^{(L)} = \langle\mathbf{t}\mathbf{t}^{\mathsf{T}}\rangle_{\text{MF}} = -\boldsymbol{\varepsilon}\boldsymbol{\varepsilon}^{\mathsf{T}} + \left[Q_f + \sigma^2 I_n\right]^{-1}, \quad (24)$$

where we define $\boldsymbol{\varepsilon} = (\mathbf{y} - \bar{\mathbf{f}})\sigma^{-2}$ and $\bar{\mathbf{t}} = \langle\mathbf{t}\rangle_{\text{MF}}$ such that

$$i\bar{\mathbf{t}} = \boldsymbol{\varepsilon} = \frac{\mathbf{y} - \bar{\mathbf{f}}}{\sigma^2} = \left[Q_f + \sigma^2 I_n\right]^{-1}\mathbf{y}. \quad (25)$$

We can also now identify the last layer of the mean-field action:

$$\mathcal{S}_{f,\text{MF}} = \frac{1}{2\sigma^2}\sum_{\mu}(f_\mu - y_\mu)^2 + \frac{1}{2}\sum_{\mu\nu} f_\mu [Q_f]^{-1}_{\mu\nu} f_\nu \quad (26)$$

Combining all the layers Eq. (19), Eq. (23), and Eq. (26), we can write the mean-field action:

$$\mathcal{S}_{\text{MF}} = \sum_{l=1}^{L-1}\mathcal{S}^{(l)}_{\text{MF}} + \mathcal{S}_{f,\text{MF}}. \quad (27)$$

This allows us to define the $\langle ... \rangle_{\text{MF}}$ with respect to the distribution:

$$\pi_{\text{MF}}(\{\mathbf{h}^{(l)}\}_{l=1}^{L-1}, \mathbf{f}) = e^{-\mathcal{S}_{\text{MF}}}/\mathcal{Z}_{\text{MF}} \quad (28)$$

and the partition function $\mathcal{Z}_{\text{MF}} = \int \prod_{l=1}^{L-1} d\mathbf{h}^{(l)} d\mathbf{f}\, \pi_{\text{MF}}(\{\mathbf{h}^{(l)}\}_{l=1}^{L-1}, \mathbf{f})$. We note that though this distribution is decoupled among layers and neurons in each

layer, it is parameterized by mean-field average quantities. These quantities are defined self consistently and, unlike the infinite width case, create both downstream and upstream dependencies between layers. These dependencies will be fleshed out in the next subsection.

1.3 Variational Gaussian approximation

Despite reducing the full DNN into a product of decoupled partition functions per neuron and layer, the resulting actions for all but the top layer, are still non-Gaussian. Following the justifications discussed in the main text, we approximate the latter using the variational Gaussian approximation (VGA), in which we search for the closest (in the KL divergence sense) Gaussian distribution, with general covariance matrix $K^{(l)}$, to the non-Gaussian mean-field distribution of the lth layer. We denote the optimal covariance matrix found for each layer $K^{(l)}$ as the pre-kernel, since it is the approximated covariance of the pre-activation $\mathbf{h}^{(l)}$. In this section, we also assume for simplicity an antisymmetric activation function, such as $\phi = \mathrm{erf}$. Due to ϕ's anti-symmetry, the DNN acquires an internal symmetry, making each pre-activation as likely as its negative. At large enough N_l, we do not expect spontaneous symmetry breaking of this internal symmetry, thus one may consider a simplified version of the VGA which involves only centered Gaussians. We note that if the activation function is not antisymmetric as in the case of ReLU, a parameter for the mean should be added to the VGA (see Sec 5 for more details).

For the first layer, one is free to choose either pre-activations or the weights themselves as the variables, as these two are linear functions of one another. Here we will use the weights, $W_{i:}^{(1)}$, (the ith row of the matrix $W^{(1)}$) and denote the covariance matrix of these weights as Σ. The pre-kernel matrix of the input layer pre-activations is then given by $K^{(1)} = X_n \Sigma X_n^{\intercal}$.

In the next two subsections, we perform the VGA to find the effective Gaussian kernel of the lth hidden layer (i.e. the pre-kernel $K^{(l)}$). Note that we apply the VGA for each layer, l where our reference distribution is the mean-field distribution we found in Sec. 1.2. The lth layer mean-field distribution is defined as $e^{-\mathcal{S}_{\text{MF}}^{(l)}} \propto \prod_j \pi_j^{(l)}$ where the distribution for each neuron j and layer l is $\pi_j^{(l)}$ and $\mathcal{S}_{\text{MF}}^{(l)}$ is defined in Eq. (19), Eq. (23), and Eq. (26). We begin with the hidden layers, turn to discuss the input and final layer, and then finally gather all the terms and report our main result which is the Equations of State (EoS)

1.3.1 $l \in [2, L-1]$

The KL divergence between the above distribution and a Gaussian distribution for all $j \in [0, N_l - 1]$ with covariance $K^{(l)}$ is then

$$
\begin{aligned}
D(\mathcal{N}(0, K^{(l)})|\pi_j^{(l)}) = &-\left\langle \frac{1}{2}\sum_{\mu\nu} h_{j\mu}^{(l)} \left[Q^{(l)}\right]^{-1}_{\mu\nu} h_{j\nu}^{(l)} \right\rangle_{K^{(l)}} \\
&- \frac{\sigma_{l+1}^2}{2N_l}\sum_{\mu\nu} A_{\mu\nu}^{(l+1)} \left\langle \phi(h_{j\mu}^{(l)})\phi(h_{j\nu}^{(l)}) \right\rangle_{K^{(l)}} + \frac{1}{2}\log\det\left(K^{(l)}\right) + \text{Const.}
\end{aligned} \tag{29}
$$

where $\langle ... \rangle_C$ indicate average with respect to a centered Gaussian distribution with covariance matrix C, and recall that $A^{(l)} = \langle \mathbf{m}^{(l)}(\mathbf{m}^{(l)})^\mathsf{T} \rangle_{\text{MF}}$. To find the pre-kernels $K^{(l)}$, we take the derivative with respect to $K^{(l)}$ and set it to zero, in order to find the closest Gaussian distribution:

$$
\begin{aligned}
&\partial_{K^{(l)}} D(\mathcal{N}(0, K^{(l)})|\pi_j^{(l)}) \\
&\qquad = -\frac{1}{2}[Q^{(l)}]^{-1} - \frac{N_{l+1}}{2N_l}\sum_{\mu\nu} A_{\mu\nu}^{(l+1)} \partial_{K^{(l)}} Q_{\mu\nu}^{(l+1)} + \frac{1}{2}[K^{(l)}]^{-1} = 0
\end{aligned} \tag{30}
$$

In the first equality, since the optimal Gaussian distribution found is the closest to the mean-field one, the expectation of the post-kernel $\tilde{Q}^{(l)}$ with respect to the Gaussian distribution is a good approximation of the mean-field expectation. This is also validated using perturbation theory in section 4. Therefore, we have that,

$$
\begin{aligned}
\sigma_{l+1}^2 \left\langle \phi(h_{j\mu}^{(l)})\phi(h_{j\nu}^{(l)}) \right\rangle_{K^{(l)}} &= \sigma_{l+1}^2 \left\langle \phi(h_{0\mu}^{(l)})\phi(h_{0\nu}^{(l)}) \right\rangle_{K^{(l)}} \\
&\approx \left\langle \tilde{Q}_{\mu\nu}^{(l+1)}(\mathbf{h}^{(l)}) \right\rangle_{\text{MF}} = Q_{\mu\nu}^{(l+1)}
\end{aligned} \tag{31}
$$

where for the last layer $\tilde{Q}^{(L)} = \tilde{Q}_f$, such that $Q^{(L)} = Q_f$. We note here that if $Q^{(l)}$ is not full rank, then $K^{(l)}$ has the same support (vector space associated with non-zero eigenvalues) as $Q^{(l)}$. We can now replace in Eq. (20) $\langle \mathbf{h}_j^{(l)}(\mathbf{h}_j^{(l)})^\mathsf{T} \rangle_{\text{MF}}$ by $K^{(l)}$ such that for $l \in [2, L-1]$:

$$
A^{(l)} \approx [Q^{(l)}]^{-1} - [Q^{(l)}]^{-1} K^{(l)} [Q^{(l)}]^{-1}. \tag{32}
$$

Note that, this is indeed the optimal distribution since taking the second derivative yields $-[K^{(l)}]^{-2}/2$ and since $K^{(l)}$ is positive definite as a covariance matrix $-[K^{(l)}]^{-2}/2$ is negative definite, therefore it is a global minimum.

1.3.2 $l = 1$

We turn to find the pre-kernel of the input layer. Here, we find it more convenient to work with the covariance matrix of the weights rather than the pre-activation, as it is a more compact object having fewer indices. We follow the same procedure

and minimize the KL divergence for all $i \in [0, N_1)$ to find the closest Gaussian distribution with covariance Σ,

$$
\begin{aligned}
D(\mathcal{N}(0,\Sigma)|\pi_i^{(1)}) = &- \left\langle \frac{d\left\|W_{i:}^{(1)}\right\|^2}{2\sigma_1^2} \right\rangle_\Sigma \\
&- \sigma_2^2 \frac{N_2}{2N_1} \sum_{\mu\nu} A_{\mu\nu}^{(2)} \left\langle \phi(h_{i\mu}^{(1)})\phi(h_{i\nu}^{(1)}) \right\rangle_\Sigma + \frac{1}{2}\log\det(\Sigma) + const \\
= &- \frac{d}{2\sigma_1^2}\mathrm{Tr}\,(\Sigma) - \frac{N_2}{2N_1} \sum_{\mu\nu} A_{\mu\nu}^{(2)} \left\langle \phi(h_{i\mu}^{(1)})\phi(h_{i\nu}^{(1)}) \right\rangle_\Sigma + \frac{1}{2}\log\det(\Sigma) + const,
\end{aligned}
$$

taking the derivative with respect to Σ

$$
\frac{\partial D(\mathcal{N}(0,\Sigma)|\pi_i^{(1)})}{\partial \Sigma} = -\frac{d}{2\sigma_1^2} I_d - \frac{N_2}{2N_1} \sum_{\mu\nu} A_{\mu\nu}^{(2)} \left[\partial_\Sigma Q^{(1)}\right]_{\mu\nu} + \frac{1}{2}\Sigma^{-1} = 0. \tag{33}
$$

where again we replace the expectation within the Gaussian optimal distribution with the mean-field expectation. By the same reasoning, we can replace in Eq. (20) the term $\langle \mathbf{h}_j^{(1)} (\mathbf{h}_j^{(1)})^\mathsf{T} \rangle_{\mathrm{MF}}$ by $K^{(2)}$, and use the result in Sec. 1.4.2, to obtain:

$$
A^{(2)} \approx [Q^{(2)}]^{-1} - [Q^{(2)}]^{-1} K^{(2)} [Q^{(2)}]^{-1}. \tag{34}
$$

Plugging in the above expression for $A^{(2)}$ and combining with Eq. (30) we have that:

$$
-\frac{d}{\sigma_1^2} I_d - \frac{N_2}{N_1} \sum_{\mu\nu} \left([Q^{(2)}]^{-1} - [Q^{(2)}]^{-1} K^{(2)} [Q^{(2)}]^{-1} \right)_{\mu\nu} \left(\partial_\Sigma Q^{(2)} \right)_{\mu\nu} + \Sigma^{-1} = 0.
$$

We note that following the VGA, one can use results of Ref. [1] to obtain explicit expressions for the post-kernels $Q_f, Q^{(l)}$. Using the definition of the pre-kernels in Eq. (31) we obtain for $\phi = \mathrm{erf}$, for all $l \in [1, L-1]$

$$
[Q^{(l+1)}]_{\mu\nu} = \sigma_{l+1}^2 \frac{2}{\pi} \sin^{-1}\left(\frac{2[K^{(l)}]_{\mu\nu}}{\sqrt{1+2[K^{(l)}]_{\mu\mu}}\sqrt{1+2[K^{(l)}]_{\nu\nu}}} \right), \tag{35}
$$

where $K^{(1)} = X_n \Sigma X_n^\mathsf{T}$ and $Q^{(L)} = Q_f$.

1.4 Equations of State (EoS)

Collecting all the results above, we obtain the following closed set of equations determining all pre-kernel and post-kernel as well as the average output of the

Langevin algorithm, $\bar{\mathbf{f}}$: namely

$$\bar{\mathbf{f}} = Q_f[\sigma^2 I_n + Q_f]^{-1}\mathbf{y} \tag{36}$$

$$[Q^{(l)}]_{\mu\nu} = \sigma_l^2 \frac{2}{\pi} \sin^{-1}\left(\frac{2[K^{(l-1)}]_{\mu\nu}}{\sqrt{1+2[K^{(l-1)}]_{\mu,\mu}}\sqrt{1+2[K^{(l-1)}]_{\nu,\nu}}}\right)$$

$$[[K^{(l-1)}]^{-1}]_{\mu\nu} = [[Q^{(l-1)}]^{-1}]_{\mu\nu} + \frac{N_l}{N_{l-1}}\mathrm{Tr}\left\{A^{(l)}\frac{\partial Q^{(l)}}{\partial[K^{(l-1)}]_{\mu\nu}}\right\} \text{ for all } l \in [2, L] \tag{37}$$

$$[\Sigma^{-1}]_{ss'} = \frac{d}{\sigma_1^2}\delta_{ss'} + \frac{N_2}{N_1}\mathrm{Tr}\left[A^{(2)}\partial_{\Sigma_{ss'}}Q^{(2)}\right] \tag{38}$$

$$A^{(l)} = [Q^{(l)}]^{-1} - [Q^{(l)}]^{-1}K^{(l)}[Q^{(l)}]^{-1} \text{ for all } l \in [2, L-1]$$

$$A^{(L)} = -(\mathbf{y}-\bar{\mathbf{f}})(\mathbf{y}-\bar{\mathbf{f}})^{\mathsf{T}}\sigma^{-4} + \left[Q_f + \sigma^2 I_n\right]^{-1}$$

where $N_L = 1$, $Q^{(L)} = Q_f$, and $K^{(1)} = X_n \Sigma X_n^{\mathsf{T}}$. While not explicitly apparent, the above expression does converge to the GP limit as $N = N_l \to \infty$ for all $l \in [2, L-1]$. Indeed, for very large N, $K^{(L-1)} = Q^{(L-1)} + O(1/N)$. Consequently, the term $[[Q^{(l)}]^{-1}(K^{(l)} - Q^{(l)})[Q^{(l)}]^{-1}]$ is $O(1/N)$. The term on the r.h.s. thus vanishes as $1/N$.

We note that the second term in Eq. (37) and Eq. (38) has a more profound meaning in terms of the information transfer between the pre-kernel and post-kernel. Looking at the KL divergence between two centered multidimensional Gaussian with kernel $K^{(l)}$ and $Q^{(l)}$ of the same dimension $m = Nn$

$$D_{\mathrm{KL}}(K^{(l)}||Q^{(l)}) = \frac{1}{2}\left(\mathrm{Tr}\left([Q^{(l)}]^{-1}K^{(l)}\right) - m + \ln\left(\frac{\det Q^{(l)}}{\det K^{(l)}}\right)\right). \tag{39}$$

Taking the derivative with respect to $K^{(l-1)}$ for $l \in [3, L-1]$ and with respect to Σ for $l = 2$, we then obtain that:

$$\begin{aligned}
&\partial_{K^{(l-1)}} D_{\mathrm{KL}}(K^{(l)}||Q^{(l)}) \\
&\quad = \frac{1}{2}\mathrm{Tr}\left(-[Q^{(l)}]^{-1}\partial_{K^{(l-1)}}Q^{(l)}[Q^{(l)}]^{-1}K^{(l)} + [Q^{(l)}]^{-1}\partial_{K^{(l-1)}}Q^{(l)}\right) \\
&= \frac{1}{2}\mathrm{Tr}\left[\left([Q^{(l)}]^{-1}(Q^{(l)} - K^{(l)})[Q^{(l)}]^{-1}\right)\partial_{K^{(l-1)}}Q^{(l)}\right] = \frac{1}{2}\mathrm{Tr}\left[A^{(l)}\partial_{K^{(l-1)}}Q^{(l)}\right],
\end{aligned} \tag{40}$$

where in the second transition we rearranged terms and used the cyclical property of the trace. Substituting this relation leads to Eq. (4) provided in the main text.

1.4.1 Extension to test points

The equations of state, as derived so far, involve only the training set. Nonetheless, they could be extended to include test points in the following straightforward

manner: Add an additional "training point" (f_*) whose MSE term looks like $(f_* - y_*)^2/2\tilde{\sigma}^2$ where $\tilde{\sigma}^2$ is some constant (rather than $(f_\mu - y_\mu)^2/2\sigma^2$), repeat the derivation, and once the EoS are obtained take $1/\tilde{\sigma}^2$ to zero.

Let us demonstrate this procedure as it applies to the 2-layer CNN presented in the main text. To this end, we consider the previously derived EoS only on $n+1$ points, the n train points ($\mu = 1..n$) and an additional test point ($*$). We next rewrite the EoS using the following block notation for $n+1$ by $n+1$ matrices

$$\begin{bmatrix} A & \mathbf{b} \\ \mathbf{b}^T & d \end{bmatrix} \tag{41}$$

with A an n by n matrix, d a scalar, and $\mathbf{b}$ a vector of size n. Similarly, $[\mathbf{a}, b]$ refers to an $n+1$ dimensional vector which is a concatenation of an n dimensional vector ($\mathbf{a}$) and the scalar variables b.

Using this notation, the first two equations in the EoS become

$$\begin{aligned}
\begin{bmatrix} \bar{\mathbf{f}} \\ f_* \end{bmatrix} &= \begin{bmatrix} Q_f & \mathbf{q} \\ \mathbf{q}^T & q_* \end{bmatrix} \begin{bmatrix} Q_f + \sigma^2 I & \mathbf{q} \\ \mathbf{q}^T & q_* + \tilde{\sigma}^2 \end{bmatrix}^{-1} \begin{bmatrix} \mathbf{y} \\ y_* \frac{\sigma^2}{\tilde{\sigma}^2} \end{bmatrix} \\
[Q_f]_{\mu\nu} &= \frac{\sigma_{\mathrm{a}}^2}{N\pi} \sum_i \sin^{-1}\left(\frac{2\mathbf{x}_{\mu,i}\Sigma\mathbf{x}_{\nu,i}}{\sqrt{1+2\mathbf{x}_{\nu,i}\Sigma\mathbf{x}_{\nu,i}}\sqrt{1+2\mathbf{x}_{\mu,i}\Sigma\mathbf{x}_{\mu,i}}} \right) \\
[\mathbf{q}]_\mu &= \frac{\sigma_{\mathrm{a}}^2}{N\pi} \sum_i \sin^{-1}\left(\frac{2\mathbf{x}_{*,i}\Sigma\mathbf{x}_{\mu,i}}{\sqrt{1+2\mathbf{x}_{*,i}\Sigma\mathbf{x}_{*,i}}\sqrt{1+2\mathbf{x}_{\mu,i}\Sigma\mathbf{x}_{\mu,i}}} \right) \\
q_* &= \frac{\sigma_{\mathrm{a}}^2}{N\pi} \sum_i \sin^{-1}\left(\frac{2\mathbf{x}_{*,i}\Sigma\mathbf{x}_{*,i}}{\sqrt{1+2\mathbf{x}_{*,i}\Sigma\mathbf{x}_{*,i}}\sqrt{1+2\mathbf{x}_{*,i}\Sigma\mathbf{x}_{*,i}}} \right)
\end{aligned} \tag{42}$$

Using standard block matrix inversion formula and the fact that $Q_f + \sigma^2 I$ and $q_* + \tilde{\sigma}^2$ are invertible we find

$$\begin{aligned}
&\begin{bmatrix} Q_f + \sigma^2 I & \mathbf{q} \\ \mathbf{q}^T & q_* + \tilde{\sigma}^2 \end{bmatrix}^{-1} = \\
&\begin{bmatrix} (Q_f + \sigma^2 I - \mathbf{q}(q_* + \tilde{\sigma}^2)^{-1}\mathbf{q}^T)^{-1} & \mathbf{0} \\ \mathbf{0}^T & (q_* + \tilde{\sigma}^2 - \mathbf{q}^T(Q_f + \sigma^2 I)^{-1}\mathbf{q})^{-1} \end{bmatrix} \\
&\begin{bmatrix} I & -\mathbf{q}(q_* + \tilde{\sigma}^2)^{-1} \\ -\mathbf{q}^T(Q_f + \sigma^2 I)^{-1} & 1 \end{bmatrix}
\end{aligned} \tag{43}$$

Next taking the limit $\tilde{\sigma}^2 \to \infty$ yields

$$\begin{aligned}
\lim_{\tilde{\sigma}^2 \to \infty} \begin{bmatrix} Q_f + \sigma^2 I & \mathbf{q} \\ \mathbf{q}^T & q_* + \tilde{\sigma}^2 \end{bmatrix}^{-1} &= \begin{bmatrix} (Q_f + \sigma^2 I)^{-1} & \mathbf{0} \\ \mathbf{0}^T & 0 \end{bmatrix} \begin{bmatrix} I & \mathbf{0} \\ -\mathbf{q}^T(Q_f + \sigma^2 I)^{-1} & 1 \end{bmatrix} \\
&= \begin{bmatrix} (Q_f + \sigma^2 I)^{-1} & \mathbf{0} \\ \mathbf{0}^T & 0 \end{bmatrix}
\end{aligned} \tag{44}$$

This simple form has several desired outcomes. First, plugged into Eq. 43 it implies a familiar GP inference formula on the train and test points. In particular, $f_* = \mathbf{q}^T K_f^{-1}\mathbf{y}$. We note that this result holds for any depth. Second, it implies that the discrepancy (ε_*) on the test point, which is given by $[Q_f + \sigma^2 I, \mathbf{q}; \mathbf{q}^T, q_* + \tilde{\sigma}^2]^{-1}[\mathbf{y}, y_*]^T$, goes to zero at $\tilde{\sigma}^2 \to \infty$. Consequently, the EoS for Σ defined on $n+1$ points with $\tilde{\sigma}^2 \to \infty$, reduce back to the original equation defined only on n training points namely

$$[\Sigma^{-1}]_{ss'} = \frac{S}{\sigma_{\mathrm{w}}^2}\delta_{ss'} - \frac{1}{C}\,\mathrm{Tr}\Big\{(\boldsymbol{\varepsilon}\boldsymbol{\varepsilon}^\mathsf{T} - {K_f}^{-1})\frac{\partial Q_f}{\partial \Sigma_{ss'}})\Big\} \tag{45}$$

Following the above, to obtain a prediction on the test point, as done in Section 2.3 in the main text, we may solve the EoS only on the train-set, obtain Σ, use that Σ in Eq. 43 to obtain Q_f and $\mathbf{q}$, and perform standard GP inference for test prediction namely $f_* = \mathbf{q}^T K_f^{-1}\mathbf{y}$.

1.4.2 Auxiliary field correlation $\langle \mathbf{m}^{(l)}(\mathbf{m}^{(l)})^\mathsf{T}\rangle_{\mathrm{MF}}$

To evaluate the second moment correlation of $\mathbf{m}^{(l)}$ for $l \in [2, L-1]$ under the mean-field action, we follow a standard field theory technique. We define the partition function and introduce a source field $J \in \mathbb{R}^{N_l \times n}$,

$$\mathcal{Z}_{\mathbf{J}}[X_n; A^{(l+1)}, Q^{(l)}] = \prod_j \mathcal{Z}_{\mathbf{J}_j} \tag{46}$$

where

$$\mathcal{Z}_{\mathbf{J}_j} = \int e^{-\sigma_{l+1}^2 \sum_{\mu\nu} A_{\mu\nu}^{(l+1)} \phi(h_{j\mu}^{(l)})\phi(h_{j\nu}^{(l)}) - \frac{1}{2}(\mathbf{J}_j + \mathbf{h}_j^{(l)})^\mathsf{T}[Q^{(l)}]^{-1}(\mathbf{J}_j + \mathbf{h}_j^{(l)})} d\mathbf{h}_j^{(l)}. \tag{47}$$

The second moment correlation then can be found by taking twice the derivative of the following free entropy and taking the source field to zero:

$$\langle \mathbf{m}^{(l)}(\mathbf{m}^{(l)})^\mathsf{T}\rangle_{\mathrm{MF}} = -\frac{1}{N_l}\sum_j \triangle_{\mathbf{J}_j} \log\left(\mathcal{Z}_{\mathbf{J}}\right)_{\mathbf{J}=0}, \tag{48}$$

the vectors $\mathbf{h}_j^{(l)}, \mathbf{J}_j \in \mathbb{R}^n$ refer to the jth column of, $\mathbf{h}^{(l)}, \mathbf{J}$ respectively. Note that, this partition function is decoupled in the neurons:

$$\begin{aligned}\triangle_J \log\left(\mathcal{Z}_{\mathbf{J}}\right) &= \sum_{kj} \nabla_{\mathbf{J}_j}^2 \log\left(\mathcal{Z}_{\mathbf{J}_k}\right) = \sum_j \nabla_{\mathbf{J}_j}\left(\mathcal{Z}_{\mathbf{J}_j}^{-1}\nabla_{\mathbf{J}_j}\mathcal{Z}_{\mathbf{J}_j}\right)\\ &= -\frac{1}{2}\sum_j \nabla_{\mathbf{J}_j}\left([Q^{(l)}]^{-1}(\mathbf{J}_j + \langle \mathbf{h}_j^{(l)}\rangle_{\mathrm{MF}}) + (\mathbf{J}_j + \langle \mathbf{h}_j^{(l)}\rangle_{\mathrm{MF}})^\mathsf{T}[Q^{(l)}]^{-1}\right)\\ &= -\frac{1}{2}\sum_j\left([Q^{(l)}]^{-1}\left(I_n + \nabla_{\mathbf{J}_j}\langle \mathbf{h}_j^{(l)}\rangle_{\mathrm{MF},J}\right) + \left(I_n + \nabla_{\mathbf{J}_j}\langle \mathbf{h}_j^{(l)}\rangle_{\mathrm{MF},J}\right)^\mathsf{T}[Q^{(l)}]^{-1}\right),\end{aligned} \tag{49}$$

where we define

$$\begin{aligned}
&\nabla_{\mathbf{J}_j} \langle \mathbf{h}_j^{(l)} \rangle_{\mathrm{MF},J} |_{\mathbf{J}=0} \\
&= \nabla_{\mathbf{J}_j} \left(\mathcal{Z}_{\mathbf{J}_j}^{-1} \int e^{-\frac{\sigma_{l+1}^2}{2} \sum_{\mu\nu} A_{\mu\nu}^{(l+1)} \phi\left(h_{j\mu}^{(l)}\right) \phi\left(h_{j\nu}^{(l)}\right) - \frac{1}{2} (\mathbf{J}_j + \mathbf{h}_j^{(l)})^\top [Q^{(l)}]^{-1} (\mathbf{J}_j + \mathbf{h}_j^{(l)})} \mathbf{h}_j^{(l)} d\mathbf{h}_j^{(l)} \right) |_{\mathbf{J}=0} \\
&\qquad = -\langle \mathbf{h}_j^{(l)} \rangle_{\mathrm{MF}} \mathcal{Z}_j^{-1} \nabla_{\mathbf{J}_j} \mathcal{Z}_{\mathbf{J}_j} |_{J=0} - \left(\langle \mathbf{h}_j^{(l)} (\mathbf{h}_j^{(l)})^\top \rangle_{\mathrm{MF}} [Q^{(l)}]^{-1} \right) \\
&\qquad\qquad = \langle \mathbf{h}_j^{(l)} \rangle_{\mathrm{MF}} \langle (\mathbf{h}_j^{(l)})^\top \rangle_{\mathrm{MF}} - \left(\langle \mathbf{h}_j^{(l)} (\mathbf{h}_j^{(l)})^\top \rangle_{\mathrm{MF}} [Q^{(l)}]^{-1} \right).
\end{aligned}$$

Note that, by symmetry for antisymmetric activation function: $\langle \mathbf{h}_j^{(l)} \rangle_{\mathrm{MF}} = \langle \mathbf{h}_j^{(l)} \rangle_{\mathrm{MF},\mathbf{J}=0} = 0$, therefore,

$$\nabla_{\mathbf{J}_j} \langle \mathbf{h}_j^{(l)} \rangle_{\mathrm{MF},J} |_{\mathbf{J}=0} = -\langle \mathbf{h}_j^{(l)} (\mathbf{h}_j^{(l)})^\top \rangle_{\mathrm{MF}} [Q^{(l)}]^{-1}.$$

Plugging this expression back in Eq. (49), and using the fact that all matrices are symmetric, we have that

$$\triangle_{\mathbf{J}} \log\left(\mathcal{Z}_{\mathbf{J}}\right) = N_l [Q^{(l)}]^{-1} \left(\langle \mathbf{h}_j^{(l)} (\mathbf{h}_j^{(l)})^\top \rangle_{\mathrm{MF}} [Q^{(l)}]^{-1} - I_n \right)$$

Plugging back in Eq. (48), we have that

$$\langle \mathbf{m}^{(l)} (\mathbf{m}^{(l)})^\top \rangle_{\mathrm{MF}} = [Q^{(l)}]^{-1} \left(I_n - \langle \mathbf{h}_j^{(l)} (\mathbf{h}_j^{(l)})^\top \rangle_{\mathrm{MF}} [Q^{(l)}]^{-1} \right).$$

1.5 An Emergent Scale

Here we identify a quantity (χ) whose scale characterizes the amount of feature learning in the DNN and its deviation from the GP limit. More specifically, when χ becomes comparable to 1, strong feature learning effects appear and perturbation theory in $1/N_l$ becomes impractical. Since χ would consist of a non-trivial combination of factors, we refer to it as an emergent scale. To define this scale, we work within our EoS and ask when Q_f changes in a noticeable manner as we lower N_l from the $N_l \to \infty$ limit at fixed σ_l^2 (the GP limit). More technically, we next solve the EoS using perturbation theory in N_l^{-1} and estimate the magnitude of the leading $O(1/N_l)$ terms we obtain.

For simplicity, we focus on a setting where the penultimate layer is linear, due to this linearity we obtain $Q_f = \sigma_L^2 K^{(L-1)}$ and so:

$$[Q_f]_{\mu\nu}^{-1} = \sigma_L^{-2} [Q^{(L-1)}]_{\mu\nu}^{-1} - \frac{1}{N_{L-1}} \left(\delta_\mu \delta_\nu - \left[Q_f + \sigma^2 I_n \right]_{\mu\nu}^{-1} \right)$$

Following the aforementioned perturbation theory in $1/N_l$, we perform a first-

order Taylor expansion of Q_f yielding

$$Q_f = \sigma_L^2 \left([Q^{(L-1)}]^{-1} - \frac{\sigma_L^2}{N_{L-1}} \left(Q^{(L-1)} \boldsymbol{\varepsilon}\boldsymbol{\varepsilon}^\intercal - Q^{(L-1)} \left[Q_f + \sigma^2 I_n \right]^{-1} \right) \right)^{-1}$$
$$= \sigma_L^2 Q^{(L-1)} + \frac{\sigma_L^2}{N_{L-1}} Q^{(L-1)} \boldsymbol{\varepsilon}\boldsymbol{\varepsilon}^\intercal Q^{(L-1)} - \frac{\sigma_L^2}{N_{L-1}} Q^{(L-1)} \left[Q_f + \sigma^2 I_n \right]^{-1} Q^{(L-1)} + O(N_{L-1}^{-2}) \tag{50}$$

To evaluate the magnitude of the first term we multiply by $\boldsymbol{\delta}$ from both sides to obtain

$$\boldsymbol{\varepsilon}^\intercal Q_f \boldsymbol{\varepsilon} = \sigma_L^2 \boldsymbol{\varepsilon}^\intercal Q^{(L-1)} \boldsymbol{\varepsilon} + \frac{\sigma_L^2}{N_{L-1}} \boldsymbol{\varepsilon}^\intercal Q^{(L-1)} \left(\boldsymbol{\varepsilon}\boldsymbol{\varepsilon}^\intercal - \left[Q_f + \sigma^2 I_n \right]^{-1} \right) Q^{(L-1)} \boldsymbol{\varepsilon} + O(N_{L-1}^{-2}) \tag{51}$$

We define $\chi = \frac{1}{N_{L-1}} \boldsymbol{\varepsilon}^\intercal Q^{(L-1)} \boldsymbol{\varepsilon}$, and find:

$$\frac{1}{N_{L-1}\sigma_L^2} \boldsymbol{\varepsilon}^\intercal Q_f \boldsymbol{\varepsilon} = \chi + \chi^2 + \frac{1}{N_{L-1}^2} \boldsymbol{\varepsilon}^\intercal Q^{(L-1)} \left[Q_f + \sigma^2 I_n \right]^{-1} Q^{(L-1)} \boldsymbol{\varepsilon} + O(N_{L-1}^{-3}) \tag{52}$$

As we argue below, the last term on the r.h.s is small compared to the first two. Putting it aside and recalling that the first term on the right-hand side is the zeroth order term in $1/N_l$, we find that χ controls the ratio between the zeroth order contribute and the first order perturbative correction. Hence, once χ becomes order 1, first-order perturbation in $1/N_l$ becomes inaccurate. However, it can be further checked that a second-order perturbation will contain a χ^3 contribution, and hence this is not just a problem in first-order perturbation theory. Rather, it is that low order perturbation theory becomes inaccurate.

Next, we argue that having non-negligible χ also implies feature learning, in the sense that Q_f changes from its $N_l \to \infty$ value. Indeed, the quantity we are examining ($\boldsymbol{\varepsilon}^\intercal Q_f \boldsymbol{\varepsilon}$) involves both the discrepancy ($\boldsymbol{\varepsilon}$) and the kernel Q_f. Thus, a-priory may change just because $\boldsymbol{\varepsilon}$ changes. However, $\boldsymbol{\varepsilon}$ is a function of Q_f via $\boldsymbol{\varepsilon} = [Q_f + \sigma^2 I]^{-1}\mathbf{y}$. Thus, it cannot undergo any change if Q_f remains inert. Thus, we conclude that a change to $\boldsymbol{\varepsilon}^\intercal Q_f \boldsymbol{\varepsilon}$ must come from a change in Q_f and hence, by our definition, from feature learning. This combined with the previous paragraph also shows that strong feature learning effects are beyond the practical reach of straightforward perturbation theory.

Following some assumptions on the support of $\boldsymbol{\varepsilon}$, the emergent scale can be presented in a more explicit manner. Indeed, assuming $\boldsymbol{\varepsilon}$ has support mainly on $Q^{(L-1)}$'s leading eigenvalues of order $\lambda_{\max}$, we can estimate $\chi \sim n^2 \text{MSE} \lambda_{\max}/N_{L-1}$, where we also use $\|\boldsymbol{\varepsilon}\|^2 = n\text{MSE}$. Last we note that χ is also a relevant scale for nonlinear penultimate layers since even in that case Q_f can be expanded in $K^{(L-1)}$ and the linear term in this expansion will yield a correction to $\boldsymbol{\varepsilon}^\intercal Q_f \boldsymbol{\varepsilon}$ proportional to the emergent scale.

We turn to estimate the magnitude of the term we neglected namely

$$\frac{1}{N_{L-1}^2} \boldsymbol{\varepsilon}^\intercal Q^{(L-1)} \left[Q_f + \sigma^2 I_n \right]^{-1} Q^{(L-1)} \boldsymbol{\varepsilon} \tag{53}$$

Noting $Q_f = \sigma_L^2 K^{(L-1)}$ and that, following our EoS., $K^{(L-1)} = Q^{(L-1)} + O(1/N_l)$, we can rewrite the above term up to $1/N_l^2$ corrections as

$$\frac{1}{N_{L-1}^2}\boldsymbol{\varepsilon}^\top Q^{(L-1)}\left[\sigma_L^2 Q^{(L-1)} + \sigma^2 I_n\right]^{-1} Q^{(L-1)}\boldsymbol{\varepsilon} \tag{54}$$

following this one can establish that

$$\frac{1}{N_{L-1}^2}\boldsymbol{\varepsilon}^\top Q^{(L-1)}\left[\sigma_L^2 Q^{(L-1)} + \sigma^2 I_n\right]^{-1} Q^{(L-1)}\boldsymbol{\varepsilon} \leq \frac{1}{N_{L-1}^2\sigma_L^2}\boldsymbol{\varepsilon}^\top Q^{(L-1)}\boldsymbol{\varepsilon} = \frac{\chi}{N_{L-1}\sigma_L^2} \tag{55}$$

which is indeed negligible compared to χ at large N_{L-1}.

1.6 Estimating Corrections to Mean-field Results

In the mean-field derivation, when focusing on the two last layers, we neglected the term

$$\frac{1}{2}\sum_{\nu\mu}\Delta[tt]_{\mu\nu}[\Delta\tilde{Q}_f(\mathbf{h}^{(L-1)})]_{\mu\nu} \tag{56}$$

where $\Delta[tt]_{\mu\nu} = t_\mu t_\nu - \langle t_\mu t_\nu\rangle_{\text{MF}}$ and $\Delta\tilde{Q}_f(\mathbf{h}^{(L-1)}) = \tilde{Q}_f(\mathbf{h}^{(L-1)}) - Q_f$. However, unlike in the case of hidden layers, where the above two "Δ" terms were both clearly a sum over many independent random variables (in the mean-field picture), here only the 2nd Δ is of that form. Hence, it is a priory less clear what makes this term negligible.

In this section, we study the effect of this term in perturbation theory and when it can be neglected. Specifically, we treat the above term as a perturbation over the mean-field limit and calculate its leading order effect on the mean-field average of, $(\mathbf{f} - \mathbf{y})/\sigma^2$ which coincides with the mean-field average of $\mathbf{t}$ which is directly related to the MSE. To expose this matter in its simplest form, we shall assume that the penultimate layer is linear. Consequently, $Q_f = \sigma_L^2 K^{(L-1)}$ and in addition, VGA becomes exact (see Eq. 17 and definition of $Q_f(\mathbf{h}^{(L-1)})$).

Turning to an action formulation, we focus on the following mean-field action of the $\mathbf{h}^{(L-1)}, \mathbf{f}$ and $\mathbf{t}$ variables together with the perturbation, namely,

$$\mathcal{S}_f = \mathcal{S}_{f,0} + \Delta\mathcal{S}_f \tag{57}$$

where

$$\begin{aligned}
\mathcal{S}_{f,0} &= i\sum_\mu t_\mu f_\mu + \frac{1}{2\sigma^2}\sum_\mu (f_\mu - y_\mu)^2 + \frac{1}{2}\sum_{\mu\nu}\langle t_\mu t_\nu\rangle_{\text{MF}}\left[\tilde{Q}_f(\mathbf{h}^{(L-1)})\right]_{\mu\nu} \qquad (58)\\
&+ \frac{1}{2}\sum_{\mu\nu} t_\mu t_\nu\left[\langle\tilde{Q}_f(\mathbf{h}^{(L-1)})\rangle_{\text{MF}}\right]_{\mu\nu}\\
\Delta\mathcal{S}_f &= \frac{1}{2}\sum_{\mu\nu}\Delta[t_\mu t_\nu]\left[\Delta\tilde{Q}_f(\mathbf{h}^{(L-1)})\right]_{\mu\nu}. \qquad (59)
\end{aligned}$$

Perturbation in the $\Delta\mathcal{S}_f$ term yields the following zeroth and first-order contributions

$$\begin{aligned}\langle t_\nu \rangle &= \langle t_\nu \rangle_{\text{MF}} \\ &+ \frac{1}{2}\sum_{a,b} \langle t_\nu \Delta[t_a t_b] \Delta\tilde{Q}_f(\mathbf{h}^{(L-1)})]_{ab} \rangle_{\text{MF,con}} + \text{Higher order terms}\end{aligned} \tag{60}$$

where $\langle ... \rangle_{\text{MF,con}}$ means connected average (i.e. one in which, following Wick's theorem, the perturbation is never contracted/averaged just with itself), and $K_f = \left[Q_f + \sigma^2 I_n\right]$. Clearly, the first order contribution is strictly zero, since by definition $\langle [\Delta\tilde{Q}_f(\mathbf{h}^{(L-1)})]_{ab} \rangle_{\text{MF}} = 0$ and due to the mean-field decoupling under $\langle .. \rangle_{\text{MF}}$, h's can only contract with themselves. We thus turn to the first non-zero contribution which occurs in second order namely

$$\frac{1}{2!4} \langle t_\nu \sum_{abde} \Delta[tt]_{ab} \Delta[tt]_{de} [\Delta\tilde{Q}_f(\mathbf{h}^{(L-1)})]_{ab} [\Delta\tilde{Q}_f(\mathbf{h}^{(L-1)})]_{de} \rangle_{\text{MF,con}}. \tag{61}$$

Noting that we are restricted to connected averages, the fact that within the mean-field picture $\mathbf{h}^{(L-1)}$'s and $\mathbf{t}$'s are uncorrelated as well as $h_i^{(L-1)}$'s between different neurons, and the fact that $\tilde{Q}_f = \sigma_L^2 N_{L-1}^{-1} \sum_{j=1}^{N_{L-1}} \mathbf{h}_j^{(L-1)} (\mathbf{h}_j^{(L-1)})^\mathsf{T}$ we obtain the following correction to $\bar{t}_\mu$

$$\frac{2}{N_{L-1}} [K_f^{-1} Q_f K_f^{-1} Q_f \bar{\mathbf{t}}]_\nu + \frac{1}{N_{L-1}} [K_f^{-1} Q_f \bar{\mathbf{t}}]_\nu \text{Tr}[K_f^{-1} Q_f] \tag{62}$$

Recalling that $K_f = Q_f + \sigma^2 I_n$, the first term in the correction to $\bar{t}_\nu$ is upper bounded by $\frac{2}{N_{L-1}} \bar{t}_\nu$ and hence negligible. The first piece of the second term is similarly upper bounded by $\frac{1}{N_{L-1}} \bar{t}_\nu$, however, it also contains a factor of $\text{Tr}[K_f^{-1} Q_f] = \sum_\lambda \sigma_L^2 \lambda / (\sigma_L^2 \lambda + \sigma^2)$, where $\sigma_L^2 \lambda$'s are eigenvalues of Q_f.

Let us discuss how these factors behave in various settings. The first settings are similar to that in the main text, only with standard rather than mean-field scaling for the top layer weight decay. Here, the $\text{Tr}[K_f^{-1} Q_f]$ term at $\sigma^2 \to 0$ is equal to n. Similarly, our upper bound for the first factor becomes exact. Together, this leads to an over n/N_{L-1} ratio between the correction to the mean-field result and the mean-field result for $\bar{t}_\mu$. Hence, when n becomes larger than, N_{L-1} we expect the mean-field treatment to fail.

Turning to nonzero σ^2, the spectrum of Q_f starts to play a role. Indeed, since the spectrum of Q_f typically decays quickly, [2] $\text{Tr}[K_f^{-1} Q_f]$ becomes proportional to the number of Q_f eigenvalues which are larger than σ^2. In addition, upper bounding $[K_f^{-1} Q_f \bar{\mathbf{t}}]_\nu$ by $\bar{t}_\nu$ may be a very loose bound if $\bar{t}$ has support on Q_f eigenvalues which are smaller than σ^2. Hence, estimating the magnitude of these corrections requires a careful case-by-case inspection. Some preliminary numerical checks we perform in teacher-student settings similar to those in the main text for $\sigma^2 \leq 0.01$ showed that the corrections to mean-field became large as soon as feature learning began. We believe that our theory still applies

to FCNs away from student-teacher settings, in particular in cases where the teacher induces a target that has support mainly on sub-leading eigenvalues of Q_f. However, we leave this matter for future study. We comment that CNNs are different in that aspect– as shown later below, they appear to obey our mean-field treatment within the standard scaling.

Last, we consider nonzero σ^2 with mean-field scaling. Here one can capitalize from the fact that the mean-field scaling, scales down Q_f, thereby making both $[K_f^{-1}Q_f\bar{\mathbf{t}}]_\nu$ and $\text{Tr}[K_f^{-1}Q_f]$ smaller. To address this issue most cleanly, let us define a GP limit to the mean-field scaling wherein we fix σ_l^2 at order 1, σ_L^2 at order ϵ (independent of N_{L-1}), and take $N_l \to \infty$. Here we find that the eigenvalues of Q_f are those of the standard GP limit, with an additional factor of ϵ. Similarly to the emergent scale section, we can now ask when the mean-field correction becomes noticeable as we reduce N_l. Assuming χ is finite but not yet of order 1, Q_f is not expected to change drastically. Hence, to obtain an estimate of the mean-field correction, we may use the $N_l \to \infty$ values for Q_f and $\bar{t}_\mu$. At small, ϵ this implies that $\text{Tr}[K_f^{-1}Q_f] \approx \text{Tr}[Q_f]/\sigma^2$ which is of the order of $n\epsilon/\sigma^2$. Similarly, $[K_f^{-1}Q_f\bar{\mathbf{t}}]_\nu$ can be estimated by $\epsilon\lambda_{\max}/\sigma^2$, where $\lambda_{\max}$ is the dominant eigenvalue of Q_f on which $\mathbf{t}$ has support. Putting this together with the N_{L-1}^{-1} factor we find that $n\lambda_{\max}\epsilon^2/(N_{L-1}\sigma^4) \ll 1$ is required which is a much less stringent requirement. Indeed, by taking ϵ to zero, one can make this as small as needed.

A key point is that making ϵ smaller makes χ bigger. Hence, our mean-field treatment works within the feature learning regime. Indeed, the emergent scale is defined as $\boldsymbol{\varepsilon}^\mathsf{T} Q^{(L-1)}\boldsymbol{\varepsilon}/N_{L-1}$ and hence does not involve ϵ directly. Decreasing ϵ can only make $\boldsymbol{\varepsilon}$ larger since $\boldsymbol{\varepsilon} = [Q_f + \sigma^2 I_n]^{-1}\mathbf{y}$. Thus, decreasing ϵ increases feature learning while making mean-field corrections more negligible. On this note, we comment that in our FCN experiments we found, similarly to Ref. [3], that mean-field scaled FCNs had better test performance compared to those which used standard scaling similar to

2 Supplementary Note - Mean-Field Equations for CNNs

In this section, we provide a sketch of the derivation of the equations of state for deep CNNs highlighting the differences between CNN and FCN. For simplicity, we follow here the derivation of a three-layer CNN. The generalization to any number of layers is straightforward. The model we consider is a three-layer CNN having two activated convolutional layers and one linear readout layer.

Specifically, we consider

$$f(\mathbf{x}) = \sum_{j=0}^{N-1} \sum_{c'=1}^{C_2} a_{c'j} \phi\left(h_{c'j}^{(2)}(\mathbf{x})\right) \tag{63}$$
$$h_{c'j}^{(2)}(\mathbf{x}) = \sum_{i=0}^{S_1-1} \sum_{c=1}^{C_1} v_{c'ci} \phi\left(\mathbf{w}_c \cdot \mathbf{x}_{i+jS_1}\right)$$

where $\mathbf{w}_c, \mathbf{x}_{i+jS_1} \in \mathbb{R}^{S_0}$, $\mathbf{a} \in \mathbb{R}^{C_2 \times N}$, $\mathbf{v} \in \mathbb{R}^{C_2 \times C_1 \times S_1}$, and the input vector $\mathbf{x} \in \mathbb{R}^d$ with $d = S_0 S_1 N$. Bold letters mark vector or tensor quantities. Similar to the FCN case, we analyze the following distribution over the pre-activations

$$p(\mathbf{f}|\mathcal{D}_n) \propto p(\mathbf{f}|X_n)\text{exp}\left(-\frac{1}{2\sigma^2}\sum_{\mu=1}^{n}\left(y_\mu - f(\mathbf{x}_\mu)\right)^2\right), \tag{64}$$

where the matrix X_n represents all the samples, $\{\mathbf{x}_\mu\}_{\mu=1}^n$, and the first term on the r.h.s. is given by

$$p(\mathbf{f}|X_n) = \left\langle \prod_\mu \delta\left[f(\mathbf{x}_\mu) - \sum_{j=0}^{N-1}\sum_{c'=1}^{C_2} a_{c'j}\phi\left(h_{c'j}^{(2)}(\mathbf{x}_\mu)\right)\right]\right\rangle_{\mathbf{a},\mathbf{v},\mathbf{w}} \tag{65}$$

where $\mathbf{f} = (f_1, ..., f_n)$ is viewed now as a random variable following the CNN outputs on all different training points, and $\langle ... \rangle_{\mathbf{a},\mathbf{v},\mathbf{w}}$ denote average over the weights $\mathbf{a}, \mathbf{v}, \mathbf{w}$. The above probability can be viewed as the prior induced on $\mathbf{f}$ by a finite random DNN with i.i.d. Gaussian weights and variance $\sigma_\text{w}^2/S_0, \sigma_\text{v}^2/(S_1C_1)$ and $\sigma_\text{a}^2/(NC_2)$ respectively for each layer. At $C_1, C_2 \to \infty$ such priors tend to a GP, however our interest here is at finite C_1, C_2.

By conditioning on the pre-activation output ($\mathbf{h}^{(2)}$), Eq. (65) can be re-written as

$$p(\mathbf{f}|X_n) = \int p(\mathbf{f}|\mathbf{h}^{(2)}, X_n)p(\mathbf{h}^{(2)}|\mathbf{w}, X_n)p(\mathbf{w})\mathcal{D}\mathbf{h}^{(2)}\mathcal{D}\mathbf{w} \tag{66}$$

where the hidden layers probabilities used above are

$$p(\mathbf{f}|\mathbf{h}^{(2)}, X_n) = \left\langle \prod_\mu \delta\left(f_\mu - \sum_{j=0}^{N-1}\sum_{c'=1}^{C_2} a_{c'j}\phi\left(h_{c'j\mu}^{(2)}\right)\right)\right\rangle_{\mathbf{a}}, \tag{67}$$
$$p(\mathbf{h}^{(2)}|\mathbf{w}, X_n) = \left\langle \prod_{\mu c' j} \delta\left(h_{c'j\mu}^{(1)} - \sum_{i=0}^{S_1-1}\sum_{c=1}^{C_1} v_{c'ci}\phi\left(\mathbf{w}_c \cdot \mathbf{x}_{\mu,i+jS_1}\right)\right)\right\rangle_{\mathbf{v}},$$

the tensor $\mathbf{h}^{(2)}$ consists of all $h_{ic\mu}^{(2)}$ the latter being the random variables describing outputs of the second layer at hidden pixel i, channel c on the $\mathbf{x}_\mu$ data-point.

Similar to the FCN, we continue our analysis by using the Fourier identity, which replaces the delta function with auxiliary fields $\mathbf{t}, \mathbf{m}$. The resulting action ($Z = \int e^{-\mathcal{S}}$) is then,

$$
\begin{aligned}
\mathcal{S} = \frac{S_0}{2\sigma_{\mathrm{w}}^2} \sum_c \|\mathbf{w}_c\|^2 - i \sum_{\mu c' j} m_{\mu c' j} h^{(2)}_{c' j}(\mathbf{x}_\mu) + \frac{1}{2} \sum_{\mu\nu j_1 j_2 c'} m_{\mu c' j_1} m_{\nu c' j_2} \tilde{Q}^{(2)}(\mathbf{w})_{\mu\nu j_1 j_2} \\
- i \sum_\mu t_\mu f_\mu + \frac{1}{2} \sum_{\mu\nu} t_\mu t_\nu \left[\tilde{Q}_f(\mathbf{h}^{(2)})\right]_{\mu\nu} + \frac{1}{2\sigma^2} \sum_\mu (f_\mu - y_\mu)^2
\end{aligned}
\tag{68}
$$

Where the "channel" post-kernels for CNN contain also summation over strides and are defined as:

$$
\tilde{Q}_f(\mathbf{h}^{(2)})_{\mu\nu} = \frac{\sigma_{\mathrm{a}}^2}{C_2 N} \sum_{jc'}^{N, C_2} \phi\left(h^{(2)}_{c' j \mu}\right) \phi\left(h^{(2)}_{c' j \nu}\right) \tag{69}
$$

$$
\tilde{Q}^{(2)}(\mathbf{w})_{\mu j_1, \nu j_2} = \frac{\sigma_{\mathrm{v}}^2}{C_1 S_1} \sum_{ic}^{S_1, C_1} \phi\left(\mathbf{w}_c \cdot \mathbf{x}_{\mu, i + j_1 S_1}\right) \phi\left(\mathbf{w}_c \cdot \mathbf{x}_{\nu, i + j_2 S_1}\right).
$$

We comment that by averaging over the auxiliary fields, $\mathbf{t}, \mathbf{m}$, one obtains the following equivalent form, containing only the pre-activations and the outputs

$$
\begin{aligned}
\mathcal{S} = \frac{S_0}{2\sigma_{\mathrm{w}}^2} \sum_c \|\mathbf{w}_c\|^2 + \frac{1}{2} \sum_{c'\mu\nu j j'} h^{(2)}_{c' j \mu} \left[\tilde{Q}^{(2)}(\mathbf{w})\right]^{-1}_{j\mu, j'\nu} h^{(2)}_{c' j' \nu} \\
+ \sum_{\mu\nu} \frac{1}{2} f_\mu \left[\tilde{Q}_f(\mathbf{h}^{(2)})\right]^{-1}_{\mu,\nu} f_\nu + \frac{1}{2\sigma^2} \sum_\mu (f_\mu - y_\mu)^2
\end{aligned}
$$

We continue with the auxiliary variables and derive the inter-layer mean-field decoupling. Where for CNN the number of channels C_1, C_2 plays the role of width in FCN. As for FCN, we note that in Eq. (68), the hidden layer and the output layer depend on their respective upstream layers only through the "channel" post-kernels. Performing our mean-field decoupling as in Sec. 1.2 we obtain the resulting action

$$
\begin{aligned}
\mathcal{S}_{\mathrm{MF}} &= \mathcal{S}^{(1)}_{\mathrm{MF}} + \mathcal{S}^{(2)}_{\mathrm{MF}} + \mathcal{S}_{f,\mathrm{MF}} \\
\mathcal{S}^{(1)}_{\mathrm{MF}} &= \frac{S_0}{2\sigma_{\mathrm{w}}^2} \sum_c \|\mathbf{w}_c\|^2 + \frac{1}{2} \sum_{\mu\nu j_1 j_2 c'} \langle \mathbf{m}\mathbf{m}^{\mathsf{T}} \rangle_{\mathrm{MF}, \mu j_1 \nu j_2} \tilde{Q}^{(2)}(\mathbf{w})_{\mu j_1 \nu j_2} \\
\mathcal{S}^{(2)}_{\mathrm{MF}} &= \frac{1}{2} \sum_{\mu\nu} \langle t_\mu t_\nu \rangle_{\mathrm{MF}} \left[\tilde{Q}_f(\mathbf{h}^{(2)})\right]_{\mu\nu} + \frac{1}{2} \sum_{\mu c' j_1, j_2} h^{(2)}_{c' j_1}(\mathbf{x}_\mu) [Q^{(2)}]^{-1}_{\mu j_1 \nu j_2} h^{(2)}_{c' j_2}(\mathbf{x}_\mu) \\
\mathcal{S}_{f,\mathrm{MF}} &= \frac{1}{2\sigma^2} \sum_\mu (f_\mu - y_\mu)^2 + \frac{1}{2} \sum_{\mu\nu} f_\mu [Q_f]^{-1}_{\mu\nu} f_\nu,
\end{aligned}
\tag{70}
$$

where the post-kernel can now be defined as the mean-field average of the "channel" post-kernel via the above mean-field action distribution:

$$[Q_f]_{\mu\nu} = \left\langle \tilde{Q}_f(\mathbf{h}^{(2)})_{\mu\nu} \right\rangle_{\mathrm{MF}} \tag{71}$$
$$[Q^{(2)}]_{\mu j,\nu j'} = \left\langle \tilde{Q}^{(2)}(\mathbf{w})_{\mu j_1,\nu j_2} \right\rangle_{\mathrm{MF}} .$$

The correlation functions similar to the FCN are, then,

$$\langle \mathbf{m}\mathbf{m}^{\mathsf{T}} \rangle_{\mathrm{MF}} = \left\langle C_2^{-1} \sum_{c',j=1}^{C_2} \mathbf{m}_{c'}\mathbf{m}_{c'}^{\mathsf{T}} \right\rangle_{\mathrm{MF}} = [Q^{(2)}]^{-1} \left(I_n - \langle \mathbf{h}_1^{(2)} (\mathbf{h}_1^{(2)})^{\mathsf{T}} \rangle_{\mathrm{MF}} [Q^{(2)}]^{-1} \right) \tag{72}$$

The last layer of auxiliary field correlation is

$$\langle t_\mu t_\nu \rangle_{\mathrm{MF}} = -\varepsilon_\mu \varepsilon_\nu + \left[(Q_f + \sigma^2 I_n)^{-1} \right]_{\mu\nu} \tag{73}$$

this is easily derive from Eq. (70). The average of $\mathbf{t}$ using the mean-field action is then,

$$i\bar{\mathbf{t}} = \boldsymbol{\varepsilon} = \frac{\mathbf{y} - \bar{\mathbf{f}}}{\sigma^2} = \left(Q_f + \sigma^2 I_n \right)^{-1} \mathbf{y}, \tag{74}$$

where we denote by bar the $\langle ... \rangle_{\mathrm{MF}}$ corresponding to averages with respect to the mean-field distribution. Note that, in the main text, we denote $\tilde{Q}^{(2)}(\mathbf{w})$ by $\tilde{Q}^{(2)}(\mathbf{h}^{(1)})$. These are the same quantities, the connection being $h_{cij}^{(1)} = \mathbf{w}_c \cdot \mathbf{x}_{i+jS_1}$.

Despite reducing the full system into decoupled systems per channel and layer, the resulting action for all but the top layer is still non-Gaussian. Following the justifications discussed in the main text and for the FCN, we approximate the latter using the variational Gaussian approximation (VGA). Assuming for simplicity an antisymmetric activation function such as erf, the CNN has an internal symmetry, making each pre-activation positive output as likely as its negative. Also, at large enough C_1, C_2 we do not expect spontaneous symmetry breaking, thus our VGA will involve only a centered Gaussian. Specifically, we denote the optimal covariance of $\mathbf{h}^{(2)}$, $K_{\mu j \nu j'}^{(2)}$ as the pre-kernel of the second layer and the optimal covariance of $\mathbf{w}$, $\Sigma_{ss'}$, is a connected to the first layer pre-kernel, $K^{(1)} = X_n \Sigma X_n^{\mathsf{T}}$.

Following the analysis in subsection. 1.3 with the above modification for CNN, we obtain the following closed set of equations determining all pre-kernels

as well as the outputs namely

$$
\begin{aligned}
\bar{\mathbf{f}} &= Q_f[\sigma^2 I_n + Q_f]^{-1}\mathbf{y} \qquad (75)\\
[Q_f]_{\mu\nu} &= \sigma_{\rm a}^2 \frac{1}{N}\sum_j \frac{2}{\pi}\sin^{-1}\left(\frac{2[K^{(2)}]_{\mu j,\nu j}}{\sqrt{1+2[K^{(2)}]_{\mu j,\mu j}}\sqrt{1+2[K^{(2)}]_{\nu j,\nu j}}}\right)\\
[Q^{(2)}]_{\mu j,\nu j'} &= \sigma_{\rm v}^2 \frac{1}{S_1}\sum_i \frac{2}{\pi}\sin^{-1}\left(\frac{2\mathbf{x}_{\mu j i}\Sigma\mathbf{x}_{\nu j' i}}{\sqrt{1+2\mathbf{x}_{\mu j i}\Sigma\mathbf{x}_{\mu j i}}\sqrt{1+2\mathbf{x}_{\nu j' i}\Sigma\mathbf{x}_{\nu j' i}}}\right)\\
[(K^{(2)})^{-1}]_{\mu j,\nu j'} &= [(Q^{(2)})^{-1}]_{\mu j,\nu j'} - \frac{1}{C_2}\mathrm{Tr}\left\{A^{(3)}\frac{\partial Q_f}{\partial [K^{(2)}]_{\mu j,\nu j'}}\right\}\\
[\Sigma^{-1}]_{ss'} &= \frac{S_0}{\sigma_{\rm w}^2}\delta_{ss'} - \frac{C_2}{C_1}\mathrm{Tr}\left[[(Q^{(2)})^{-1}(K^{(2)}-Q^{(2)})(Q^{(2)})^{-1}]\partial_{\Sigma_{ss'}}Q^{(2)}\right]\\
A^{(3)} &= (\mathbf{y}-\bar{\mathbf{f}})(\mathbf{y}-\bar{\mathbf{f}})^{\mathsf{T}}\sigma^{-4} - \left[Q_f+\sigma^2 I_n\right]^{-1}
\end{aligned}
$$

2.1 An Emergent Scale in CNNs

Following the FCN case, we again examine the EoS for the penultimate layer assuming that it is linear, solve them using perturbation theory in $1/C_l$, and use the magnitude of the correction to estimate the scale at which feature learning becomes important. For our CNNs we have that $[Q_f]_{\mu\nu} = \sigma_L^2 N^{-1}\sum_j K^{(L-1)}_{\mu j,\nu j}$ which yields the following equation of state for $K^{(L-1)}$

$$
[K^{(L-1)}]^{-1}_{\mu i,\nu j} = [Q^{(L-1)}]^{-1}_{\mu i,\nu j} - \frac{\delta_{ij}}{C_{L-1}N}\left(\varepsilon_\mu\varepsilon_\nu - \left[Q_f+\sigma^2 I_n\right]^{-1}_{\mu\nu}\right)
$$

Next, we perform a leading order perturbation theory in $1/C_l$ (or equivalently in the second term on the r.h.s) yielding

$$
\begin{aligned}
[K^{(L-1)}]_{\mu i,\nu j} = [Q^{(L-1)}]_{\mu i,\nu j} &+ \frac{1}{C_{L-1}N}\sum_{kab}[Q^{(L-1)}]_{\mu i,ak}\varepsilon_a\varepsilon_b[Q^{(L-1)}]_{bk,\nu j}\\
&- \frac{1}{C_{L-1}N}\sum_{kab}[Q^{(L-1)}]_{\mu i,ak}[Q_f+\sigma^2 I_n]^{-1}_{ab}[Q^{(L-1)}]_{bk,\nu j} + O(1/(C_{L-1}^2))
\end{aligned}
$$

As justified in the FCN case, we focus on the second term on the r.h.s. and look again at $\boldsymbol{\varepsilon}^{\mathsf{T}}Q_f\boldsymbol{\varepsilon}$ yielding,

$$
\boldsymbol{\varepsilon}^{\mathsf{T}}Q_f\boldsymbol{\varepsilon} = \frac{\sigma_L^2}{N}\sum_{i\mu\nu}\varepsilon_\mu Q^{(L-1)}_{\mu i,\nu i}\varepsilon_\nu + \frac{\sigma_L^2}{C_{L-1}N^2}\sum_{ikab\mu\nu}\varepsilon_\mu[Q^{(L-1)}]_{\mu i,ak}\varepsilon_a\varepsilon_b[Q^{(L-1)}]_{bk,\nu i}\varepsilon_\nu \qquad (76)
$$

We define the ratio of the second to the first term as the emergent scale. Notably, for $N=1$ it coincides with the definition for FCNs. In addition, considering the 2-layer CNN studied in the main text and estimating it using the same approximations, it yields the same scale cited in the main text.

2.2 Estimating Mean-field corrections - CNNs

In the FCN case, we found that an additional ingredient, on top of large N_l, is needed for our mean-field decoupling to hold - either mean-field scaling or a target with support only on weak Q_f eigenvalues. For the CNNs we have studied, and quite possibly for a much larger family of CNNs, this additional ingredient comes naturally from the read-out layer averages over N latent pixels in the penultimate layers. Since these are expected to be somewhat independent, one can hope that summing over these terms is similar to increasing the number of channels by a factor of N (the number of pixels in the penultimate layer). Here we establish this more concretely. Similar to the FCN we calculate the average of the discrepancy t_μ up to the second order:

$$\langle t_\mu \rangle = \langle t_\mu \rangle_{\text{MF}} + \frac{1}{2!4} \langle t_\mu \sum_{abde} \Delta[tt]_{ab} \Delta[tt]_{de} [\Delta\tilde{Q}_f(\mathbf{h}^{(L-1)})]_{ab} [\Delta\tilde{Q}_f(\mathbf{h}^{(L-1)})]_{de} \rangle_{\text{MF,con}} + \text{Higher order terms} \quad (77)$$

For CNN, second order term can be further simplified by using Wick theorem for pre-activations of the last layer when again we consider the case of linear activation function

$$\frac{\sigma_L^4}{8N^2 C_{L-1}} \sum_{ji} \sum_{abde} \langle t_\mu \left(t_a t_b - \langle t_a t_b \rangle_{\text{MF}} \right) \left(t_d t_e - \langle t_d t_e \rangle_{\text{MF}} \right) \left(K_{ajei}^{(L-1)} K_{bjdi}^{(L-1)} + K_{ajdi}^{(L-1)} K_{bjei}^{(L-1)} \right) \rangle_{\text{MF,con}} \quad (78)$$

A similar derivation to 1.6 yields the following correction to $\bar{t}_\mu$ $((f_\mu - y_\mu)/\sigma^2)$

$$\frac{2\sigma_L^4}{C_{L-1}N^2} \sum_{abdeij} [K_f^{-1}]_{\mu a} K_{ai,bj}^{(L-1)} [K_f^{-1}]_{bd} K_{di,ej}^{(L-1)} \bar{t}_e \quad (79)$$

$$+ \frac{\sigma_L^4}{C_{L-1}N^2} \sum_{ij} \left(\sum_{ab} [K_f^{-1}]_{\mu a} K_{ai,bj}^{(L-1)} \bar{t}_b \right) \left(\sum_{ab} [K_f^{-1}]_{ab} K_{ia,jb}^{(L-1)} \right)$$

Unlike in the FCN case, $K^{(L-1)} \in \mathbb{R}^{Nn \times Nn}$ is not proportional to $[K_f]_{\mu\nu} = \sigma^2 \delta_{\mu\nu} + \sigma_L^2 N^{-1} \sum_j K_{\mu j,\nu j}^{(L-1)}$ where $\mu, \nu \in [1, n]$, in the $\sigma^2 \to 0$ limit. In particular, for $N > 1$, the two matrices have different dimensions. Hence, we cannot cancel them together just yet. To obtain an order of magnitude estimate of the second (and more dominant) term in this perturbation, we turn to a different route and compare the mean-field value of the norm, $\sum_\mu |\langle t_\mu \rangle|^2$ which is $\sum_\mu \delta_\mu^2$ with its leading correction. Following similar arguments to the FCN section, we find that the correction is dominated by

$$\frac{\sigma_L^4}{C_{L-1}N^2} \sum_{ij} \left(\sum_{\mu ab} \bar{t}_\mu [K_f^{-1}]_{\mu a} K_{ai,bj}^{(L-1)} \bar{t}_b \right) \left(\sum_{ab} [K_f^{-1}]_{ab} K_{ia,jb}^{(L-1)} \right) \quad (80)$$

To simplify this expression, we next note that for data-sets in which for each $\mathbf{x}_\mu$ there exists a "symmetry-partner" point wherein all coordinates in the fan-in

of the i'th latent pixels are flipped - the second, trace-like, term must vanish for $i \neq j$. This is due to the fact that K_f is invariant under the action of the associated symmetry, whereas $K^{(L-1)}_{*i,*j}$ receives a minor sign whenever $i \neq j$. As $n \to \infty$ we expect this symmetry to be approximately realized as it is a symmetry of the underlying measure from which $\mathbf{x}_\mu$ are drawn. Following this, we remove $i \neq j$ terms from the summation.

Next we notice that due to the approximate translation symmetry of the dataset, at large n

$$\sum_{ab} [K_f^{-1}]_{ab} K^{(L-1)}_{ia,ib} \tag{81}$$

becomes independent of i. We thus replace it by its average and perform the remaining summation over, i which now involves only the first term to obtain

$$\frac{1}{C_{L-1}N} \left(\sum_{\mu ab} \bar{t}_\mu [K_f^{-1}]_{\mu a} [Q_f]_{ab} \bar{t}_b \right) \left(\sum_{ab} [K_f^{-1}]_{ab} [Q_f]_{ab} \right) \tag{82}$$

Recall that $[Q_f]_{\mu\nu} = \sigma_L^2 N^{-1} \sum_j K^{(L-1)}_{\mu j,\nu j}$. This resulting expression is very similar to its FCN version (with standard scaling) with one crucial difference, which is the appearance of the aforementioned $1/N$ factor. More specifically, the first summation is smaller than the zeroth term ($\sum_\mu t_\mu^2$). The scale controlling the mean-field decoupling is therefore $\frac{1}{C_{L-1}N} \operatorname{Tr}\left[K_f^{-1} Q_f\right]$. Thus, at large N, we can have a reliable mean-field decoupling even when $n = C_{L-1}$.

3 Supplementary Note - Toy Example - One Hidden Layer

In this section, we provide a detailed calculation of the toy example presented in the main text (subsection 2.3). We consider $\mathbf{x}_\mu$'s as a center Gaussian vector of size NS with random i.i.d. entries with zero mean and variance 1. We choose a target of the form $y(\mathbf{x}) = \sum_i a_i^* \mathbf{w}^* \cdot \mathbf{x}_i$. The activation function here is $\phi = \text{erf}$. Specifically, we consider a student network of the form

$$f(\mathbf{x}_\mu) = \sum_{i=1}^{N} \sum_{c=1}^{C} a_{ic} \, \text{erf}\left(\mathbf{w}_c \cdot \mathbf{x}_{\mu,i}\right). \tag{83}$$

requiring enough data-points to resolve the target sets $n > N + S$ (number of target parameters) while staying within the over-parameterized regime implies $n < CNS$. We further consider the large-scale "thermodynamic" limit, where $C, S, N, n \gg 1$.

Similarly to the 3 layer case, the equations of states here are given by,

$$
\begin{aligned}
[\Sigma^{-1}]_{ss'} &= \frac{S}{\sigma_{\mathrm{w}}^2}\delta_{ss'} + \frac{1}{C}\sum_{\mu\nu} A^{(2)}_{\mu\nu}\frac{\partial [Q_f]_{\mu\nu}}{\partial \Sigma_{ss'}} \\
[Q_f]_{\mu\nu} &= \frac{2\sigma_{\mathrm{a}}^2}{\pi N}\sum_i \sin^{-1}\left(\frac{2\mathbf{x}_{\mu,i}\Sigma\mathbf{x}_{\mu,i}}{\sqrt{1+2\mathbf{x}_{\mu,i}\Sigma\mathbf{x}_{\mu,i}}\sqrt{1+2\mathbf{x}_{\nu,i}\Sigma\mathbf{x}_{\nu,i}}}\right) \\
\boldsymbol{\varepsilon} &= [Q_f + \sigma^2 I_n]^{-1}\mathbf{y} \\
A^{(2)}_{\mu\nu} &= -\varepsilon_\mu\varepsilon_\nu + \left[(Q_f+\sigma^2 I_n)^{-1}\right]_{\mu\nu}
\end{aligned}
\tag{84}
$$

These can be viewed as non-linear equations in the $S(S-1)/2$ variables making up the symmetric matrix Σ. Having these variables determines the t_μ variables directly.

We begin with approximating the GP inference appearing in the last equation. This can be represented as $\boldsymbol{\varepsilon} = [\mathbf{y} - \bar{\mathbf{f}}]/\sigma^2$ where, $\bar{\mathbf{f}} = Q_f[Q_f + \sigma^2 I_n]^{-1}\mathbf{y}$ following the standard GP prediction formula (for the training set). At large n, $\bar{\mathbf{f}}$ can be approximated using the equivalence kernel (EK) approximation [4] together with its perturbative corrections [5, 6] (non-perturbative approaches in $1/n$ could also be considered [7, 8]). Within this approximation scheme, one considers $[Q_f]_{\mu\nu} = Q_f(\mathbf{x}_\mu, \mathbf{x}_\nu)$ as the continuum operator $Q_f(\mathbf{x}, \mathbf{y})$, diagonalized on the data-set measure ($d\mu$), leading to the following formula for $\bar{f}(\mathbf{x})$ in the strict EK limit

$$
\bar{f}(\mathbf{x}) = \sum_\lambda \frac{\lambda}{\lambda + \sigma^2/n} y_\lambda \phi_\lambda(\mathbf{x}), \tag{85}
$$

where $\lambda, \phi_\lambda(\mathbf{x})$ are the eigenvalues and eigenfunctions of Q_f and $y_\lambda = \int d\mu_x y(\mathbf{x})\phi_\lambda(\mathbf{x})$. To obtain an explicit formula for, $\bar{t}(\mathbf{x}) = [y(\mathbf{x}) - \bar{f}(\mathbf{x})]/\sigma^2$, we proceed by solving the eigenvalue problem. To this end, we first consider the kernel action on a general linear function $(\mathbf{w'} \cdot \mathbf{z}_j)$, where $\mathbf{z}_j, \mathbf{w'}$ are vectors of size S for all j, and $\mathbf{z}_j$ is drawn from the dataset measure,

$$
\int d\mu_z Q_f(\mathbf{x}, \mathbf{z})\mathbf{w}' \cdot \mathbf{z}_j = \int d\mu_z \frac{\sigma_{\mathrm{a}}^2}{N}\sum_i \int d\mathbf{w} p(\mathbf{w}) \operatorname{erf}(\mathbf{w}\cdot\mathbf{x}_i)\operatorname{erf}(\mathbf{w}\cdot\mathbf{z}_i)(\mathbf{w}'\cdot\mathbf{z}_j). \tag{86}
$$

where $p(\mathbf{w})$ is a centered Gaussian with covariance matrix Σ. In the second transition, we undo the kernel integral, where $d\mu_z$ is a Gaussian measure. We then exchange the order of integration and sum over the weights and the data. We now do the integration over the data,

$$
\int d\mu_z \operatorname{erf}(\mathbf{w}\cdot\mathbf{z}_i)\mathbf{w}'\cdot\mathbf{z}_j = \delta_{ij}\sqrt{\frac{4}{\pi}}\frac{\mathbf{w}'\cdot\mathbf{w}}{\sqrt{1+2\|\mathbf{w}\|^2}} \quad \approx \delta_{ij}\sqrt{\frac{4}{\pi}}\frac{\mathbf{w}'\cdot\mathbf{w}}{\sqrt{1+2\langle\|\mathbf{w}\|^2\rangle_\Sigma}} \tag{87}
$$

where on the r.h.s. we noted that as $S \gg 1$, $\|\mathbf{w}\|^2$ is weakly fluctuating and close to its mean. Next, we perform the $\int d\mathbf{w}p(\mathbf{w})$ integral which, following this mean-field replacement, is now of the same type as the previous one. Overall this yields

$$\int d\mu_z \frac{\sigma_a^2}{N} \sum_i \int d\mathbf{w}p(\mathbf{w}) \operatorname{erf}(\mathbf{w} \cdot \mathbf{x}_i) \operatorname{erf}(\mathbf{w} \cdot \mathbf{z}_i)(\mathbf{w'} \cdot \mathbf{z}_j) \tag{88}$$

$$= \frac{\sigma_a^2}{N} \sum_i \sqrt{\frac{4}{\pi}} \frac{1}{\sqrt{1 + 2\langle\|\mathbf{w}\|^2\rangle_\Sigma}} \int d\mathbf{w}p(\mathbf{w}) \operatorname{erf}(\mathbf{w} \cdot \mathbf{x}_j)(\mathbf{w}' \cdot \mathbf{w})$$

$$= \frac{\sigma_a^2}{N} \sqrt{\frac{4}{\pi}} \frac{1}{\sqrt{1 + 2\langle\|\mathbf{w}\|^2\rangle_\Sigma}} \sqrt{\frac{4}{\pi}} \frac{\mathbf{w}'\Sigma\mathbf{x}_j}{\sqrt{1 + 2\mathbf{x}_j^\intercal \Sigma \mathbf{x}_j}}$$

Using again $S \gg 1$ we replace $2\mathbf{x}_j^\intercal \Sigma \mathbf{x}_j$ by its mean under $\int d\mu_x$. Following this, we obtain that the action of Q_f preserves the space of linear function. Furthermore, we see that diagonalizing Q_f in this subspace reduces to diagonalizing Σ. We thus reach the conclusion that since $y(\mathbf{x})$ is a linear function, so must be $\bar{f}(\mathbf{x})$ and hence $\bar{t}(\mathbf{x})$.

We turn to the quantity $\sum_{\mu\nu} A_{\mu\nu}^{(2)} \frac{\partial [Q_f]_{\mu\nu}}{\partial \Sigma_{ss'}}$ appearing in the equation of state for Σ. For the moment, we omit fluctuation piece of $A_{\mu\nu}^{(2)}$ and replace it by $-\varepsilon_\mu \varepsilon_\nu$. Below, we will show that the contribution of this fluctuation term is negligible at large, n which is our focus here. Following this, we approximate the two summations with the following expression

$$\sum_{\mu\nu} \varepsilon_\nu \varepsilon_\mu [Q_f]_{\mu\nu} \approx n^2 \int \int d\mu_x d\mu_y \varepsilon(\mathbf{x}) \varepsilon(\mathbf{y}) Q_f(\mathbf{x}, \mathbf{y}) \tag{89}$$

Next, we argue that $\varepsilon(\mathbf{x}) = \sum_i b_i(\mathbf{w}^* \cdot \mathbf{x}_i)$ at large n, where b_i's are some real numbers. Indeed, at large n when replacing summations by integrals and all matrices by their continuum kernels, the full symmetry of the measure from which $\mathbf{x}_\mu$'s are drawn becomes manifest in the equations. The latter amounts to an independent orthogonal rotation ($O(S)$) of each, $\mathbf{x}_i$ which leaves $\mathbf{w}^*$ invariant. Recalling the previous result, that $\bar{f}(\mathbf{x})$ is linear, together with this symmetry, implies that $\bar{f}(\mathbf{x}) = \sum_i c_i(\mathbf{w}^* \cdot \mathbf{x}_i)$ where c_i's are some real numbers. Consequently, $\varepsilon(\mathbf{x}) = \sigma^{-2}[y(\mathbf{x}) - \bar{f}(\mathbf{x})]$ is of the same form.

Using the above ansatz for, $\bar{t}(\mathbf{x})$ we can solve for the r.h.s. of Eq. (89). To this end, we again rewrite the r.h.s. by undoing the kernel integral and exchanging the order of the integration and the sum,

$$n^2 \int \int d\mu_x d\mu_y \varepsilon(\mathbf{x}) \varepsilon(\mathbf{y}) \frac{\sigma_a^2}{N} \sum_i \int d\mathbf{w}p(\mathbf{w}) \operatorname{erf}(\mathbf{w} \cdot \mathbf{x}_i) \operatorname{erf}(\mathbf{w} \cdot \mathbf{y}_i) \tag{90}$$

$$= n^2 \frac{\sigma_a^2 \|\mathbf{b}\|^2}{N} \frac{4}{\pi} \int d\mathbf{w}p(\mathbf{w}) \frac{(\mathbf{w}^* \cdot \mathbf{w})^2}{1 + 2\|\mathbf{w}\|^2}$$

where we used the fact that

$$\left[\int d\mu_x \varepsilon(\mathbf{x})\,\mathrm{erf}(\mathbf{w}\cdot\mathbf{x}_i)\right]^2 = \frac{4}{\pi}\frac{(\mathbf{u}_i\cdot\mathbf{w})^2}{1+2\|\mathbf{w}\|^2}, \tag{91}$$

where $\mathbf{u}_i = b_i\mathbf{w}^*$.

Next we use again the fact that $\|\mathbf{w}\|^2$ is weakly fluctuating at large S to perform the remaining integration over $\mathbf{w}$ and obtain

$$n^2\frac{\sigma_{\mathrm{a}}^2\|\mathbf{b}\|^2}{N}\frac{4}{\pi}\frac{(\mathbf{w}^*)^{\mathsf{T}}\Sigma\mathbf{w}^*}{1+2\langle\|\mathbf{w}\|^2\rangle_\Sigma} \tag{92}$$

Here one can also see a different justification for the VGA underlying our equations of state. Indeed, Eq. (89) is exactly the non-linear term in the mean-field-decoupled-action for the input layer. At large, n it leads to Eq. 90 where replacing $\|\mathbf{w}\|^2$ by its expectation value makes the term quadratic. As the first term in that action is quadratic in, $\mathbf{w}$ the overall action becomes Gaussian, as the VGA assumes.

Following the above simplification, the equation for Σ becomes

$$\Sigma^{-1} = \frac{S}{\sigma_{\mathrm{w}}^2}I_S - \frac{4\|\mathbf{b}\|^2 n^2\sigma_{\mathrm{a}}^2}{CN(1+2\,\mathrm{Tr}[\Sigma])\pi}\mathbf{w}^*(\mathbf{w}^*)^{\mathsf{T}} \tag{93}$$

revealing that only the eigenvector along $\mathbf{w}^*$ is affected by training. At large, S we may thus replace $\mathrm{Tr}[\Sigma]$ by σ_{w}^2 rendering the above an explicit formula for Σ.

Next, we return to the eigenvalue equation for Q_f (Eq. 88) and use the fact that $\mathbf{w}^*$ is an eigenvalue (l_*) of Σ

$$\begin{aligned}\int d\mu_z Q_f(\mathbf{x},\mathbf{z})y(\mathbf{z}) &= \lambda_y y(\mathbf{x}) \\ \lambda_y &= \frac{\sigma_{\mathrm{a}}^2}{N}\frac{4}{\pi}\frac{1}{1+2\sigma_{\mathrm{w}}^2}l_* \\ l_* &= \left[\frac{S}{\sigma_{\mathrm{w}}^2} - \frac{4n^2\sigma_{\mathrm{a}}^2}{CN(1+2\sigma_{\mathrm{w}}^2)\pi}\|\mathbf{b}\|^2\|\mathbf{w}^*\|^2\right]^{-1}\end{aligned} \tag{94}$$

Taking next the EK predictions along with its leading correction yields

$$\bar{f}(\mathbf{x}) = q_{\mathrm{train}}\frac{\lambda_y}{\lambda_y+\sigma^2/n}y(\mathbf{x}) \tag{95}$$

where q_{train} is 1 at the strict equivalence kernel limit. Taking the leading order correction as in Refs. [5, 9] one finds

$$\begin{aligned}q_{\mathrm{train}} &= \frac{(1-\alpha_{\mathrm{EK}}(1-\sigma^{-2}\bar{C}_n\alpha_{\mathrm{EK}}))}{(1-\alpha_{\mathrm{EK}})} \\ \alpha_{\mathrm{EK}} &= \frac{\sigma^2/n}{\lambda_y+\sigma^2/n}\end{aligned} \tag{96}$$

where $\bar{C}_n$ is the posterior covariance in the EK limit given by $\sum_\lambda \frac{1}{\lambda^{-1}+n/\sigma^2}$, where λ are $Q_f(\mathbf{x},\mathbf{y})$'s eigenvalues in the $C \to \infty$ limit. In practice, we estimated $\bar{C}_n$ numerically by diagonalizing large kernels. For $N, S = 20, 64$ found $\bar{C}_{800} = 0.13$ and $\bar{C}_{1600} = 0.078$.

Notably since $\varepsilon(\mathbf{x})$ came out proportional to $y(\mathbf{x})$ we obtained a simplified form for $\varepsilon(\mathbf{x})$ containing only a single free parameter (α)

$$\varepsilon(\mathbf{x}) = \alpha y(\mathbf{x}) \tag{97}$$

In addition we may now replace all the $\|\mathbf{b}\|^2$ factor appearing above with $\alpha^2\|\mathbf{a}^*\|^2$.

Altogether, this yields the following non-linear equation for the scalar quantity α

$$\sigma^2\alpha = 1 - \frac{q_{\text{train}}\lambda_\infty\left[1 - \frac{\|\mathbf{a}^*\|^2\|\mathbf{w}^*\|^2\sigma_{\text{w}}^2\sigma_{\text{a}}^2(n\alpha)^2}{NSC}\frac{4}{(1+2\sigma_{\text{w}}^2)\pi}\right]^{-1}}{\lambda_\infty\left[1 - \frac{\|\mathbf{a}^*\|^2\|\mathbf{w}^*\|^2\sigma_{\text{w}}^2\sigma_{\text{a}}^2(n\alpha)^2}{NSC}\frac{4}{(1+2\sigma_{\text{w}}^2)\pi}\right]^{-1} + \sigma^2/n} \tag{98}$$

$$\lambda_\infty = \frac{4\sigma_{\text{a}}^2\sigma_{\text{w}}^2}{\pi(1+2\sigma_{\text{w}}^2)NS}$$

Solving the above equation for α, one obtains Σ and Q_f using the equations of state. Using, Q_f we can calculate the DNNs predictions on the test set using standard GP inference. We note by passing that one can also estimate the result of this GP inference using EK. However, we found that just keeping a leading perturbative correction to the EK result, as done for the training set, resulted in $20-30\%$ discrepancies when compared to exact the GP inference formula. As estimating GP inference was not a main focus of the current work, we instead opted to perform this last GP inference on the test set numerically. In principle, other analytical methods for estimating GP inference could be used here [2]. Finally, we note that when estimating α_{train} and α_{test} on a specific dataset they are defined as

$$\alpha_{\text{train}} = \frac{\sum_{\mu\in\text{Train}}(y_\mu - f_\mu)y_\mu}{\sum_{\mu\in\text{Train}} y_\mu^2} \tag{99}$$

$$\alpha_{\text{test}} = \frac{\sum_{\mu\in\text{Test}}(y_\mu - f_\mu)y_\mu}{\sum_{\mu\in\text{Test}} y_\mu^2} \tag{100}$$

where *Train* and *Test* refer to samples taken from the train and test datasets, respectively.

Last we turn to discuss the fluctuation term we omitted given by

$$C^{-1}\sum_{\mu\nu}\left[Q_f + \sigma^2 I_n\right]^{-1}_{\mu\nu}\frac{\partial[Q_f]_{\mu\nu}}{\partial\Sigma_{ss'}} \tag{101}$$

We wish to compare its contribution to that of $C^{-1}\,\text{Tr}\left[\boldsymbol{\varepsilon}\boldsymbol{\varepsilon}^\mathsf{T}\frac{\partial[Q_f]_{\mu\nu}}{\partial\Sigma_{ss'}}\right]$. To this end, we write Q_f in terms of its eigenvectors ($\mathbf{v}_k$) and eigenvalues (λ_k)

$$C^{-1}\left[Q_f + \sigma^2 I_n\right]^{-1} = C^{-1}\sum_k \frac{\mathbf{v}_k\mathbf{v}_k^\mathsf{T}}{\lambda_k + \sigma^2} \tag{102}$$

aiming for an order of magnitude estimation, we perform the following two approximations: First, we approximate the eigenvalue by the leading n eigenvalues of the continuum kernel time n. Second, we take this continuum kernel to be GP kernel. The latter is justified by the fact that the feature-learning effects we found are large, but still do not correspond to an order of magnitude change. Following this, we obtain NS degenerate eigenvalues equal to $n\lambda_\infty$ and the corresponding $\mathbf{v}_k$'s span all linear functions on input space (sampled on the training set).

Next, we estimate how adding such terms to the previous computation affects the equation for Σ^{-1}. First, we note that $n\lambda_\infty \sim n/(NS) \sim 1$, in our two experiments. Next, we imagine repeating the computation of the previous section, with these extra terms corresponding to the various different $\mathbf{v}_k \mathbf{v}_k^\mathsf{T}$. Notably each such term would enter the computation in the same exact manner to $\bar{\mathbf{t}}$ (see Eq. 89) namely

$$\sum_{\mu\nu} [\mathbf{v}_k]_\nu [\mathbf{v}_k]_\mu [Q_f]_{\mu\nu} \approx n \int\int d\mu_x d\mu_y v_k(\mathbf{x}) v_k(\mathbf{y}) Q_f(\mathbf{x}, \mathbf{y}) \tag{103}$$

the only two differences are that (i) $||\mathbf{t}||^2 = \alpha^2 n$ whereas $||[\mathbf{v}_k]||^2 = 1$ (hence a factor n on the r.h.s. was lost compared to Eq. 89) and (ii) $\mathbf{v}_k$ can have any dependence on $\mathbf{x}_i$ and not only through $\mathbf{w}^* \cdot \mathbf{x}_i$. For concreteness, let us span the continuum version of these $\mathbf{v}_k$ by $[\mathbf{x}_i]_s$. Summed together, all these NS eigenvalues will end up augmenting the r.h.s. Eq. 93 into

$$\Sigma^{-1} = \frac{S}{\sigma_\mathrm{w}^2} I_S - \frac{4\|\mathbf{b}\|^2 n^2 \sigma_\mathrm{a}^2}{CN(1 + 2\,\mathrm{Tr}[\Sigma])\pi} \mathbf{w}^* (\mathbf{w}^*)^\mathsf{T} + \frac{1}{n\lambda_\infty + \sigma^2} \frac{4n\sigma_\mathrm{a}^2}{C(1 + 2\,\mathrm{Tr}[\Sigma])\pi} I_S \tag{104}$$

comparing the first and last term on the r.h.s we find it is negligible for $CS(n\lambda_\infty + \sigma^2) \gg n$. Notably, even for our $n = 800, S = 64$ experiment at $C = 80$, we find this last term is negligible.

Last, we note that we solved the equations of states numerically both with and without the extra fluctuation ($[Q_f + \sigma^2 I]^{-1}$) term and found a negligible difference. For instance, α_train for the $n = 800$ experiment with $C = 80$ came out to be 0.384 (0.377) without (with) the fluctuation term. Similarly, for, $n = 1600, C = 640$ we found 0.375 (0.370) without (with) the fluctuation term.

3.1 Continuum Limit of Summations for CNNs and FCNs

Here, we argue that the replacement involved in Eq. (89) is valid for $n \gg \sqrt{NS}$. We further comment on some implications this has for the fully-connected case ($N = 1$) with standard scaling. To show this, we consider a summation of the form

$$\frac{1}{n^2} \sum_{\mu\nu} \varepsilon_\mu \varepsilon_\nu [Q_f]_{\mu\nu} = \frac{1}{n^2} \sum_{\mu\nu, ii'} (\mathbf{u}_i \cdot [\mathbf{x}_\mu]_i)(\mathbf{u}_{i'} \cdot [\mathbf{x}_\nu]_{i'}) [Q_f]_{\mu\nu} \tag{105}$$

undoing the kernel integral as we have done before (see for example Eq. (86)) yields

$$\sum_{\mu\nu} \frac{\sigma_{\mathrm{a}}^2}{Nn^2} \sum_{jii'} \int d\mathbf{w} p(\mathbf{w}) (\mathbf{u}_i \cdot [\mathbf{x}_\mu]_i)(\mathbf{u}_{i'} \cdot [\mathbf{x}_\nu]_{i'}) \operatorname{erf}(\mathbf{w} \cdot [\mathbf{x}_\mu]_j) \operatorname{erf}(\mathbf{w} \cdot [\mathbf{x}_\nu]_j) \tag{106}$$

For simplicity, we present the analysis for $N = 1$, and report the results for general N by symmetry. Re-focusing on the relevant summation,

$$I = \frac{1}{n^2} \sum_{\mu\nu} (\mathbf{u} \cdot \mathbf{x}_\mu)(\mathbf{u} \cdot \mathbf{x}_\nu) \operatorname{erf}(\mathbf{w} \cdot \mathbf{x}_\mu) \operatorname{erf}(\mathbf{w} \cdot \mathbf{x}_\nu), \tag{107}$$

we consider the average (denoted below by a bar) and variance of I over the dataset, $\{\mathbf{x}_\mu\}_{\mu=1}^n$ where each sample is drawn from the measure $d\mu_x$, conditioning on the values of $\mathbf{w}$ and $\mathbf{u}$. This yield,

$$\bar{I} = \frac{1}{n^2} \sum_{\mu\nu} \mathbb{E}\left[(\mathbf{u} \cdot \mathbf{x}_\mu)(\mathbf{u} \cdot \mathbf{x}_\nu) \operatorname{erf}(\mathbf{w} \cdot \mathbf{x}_\mu) \operatorname{erf}(\mathbf{w} \cdot \mathbf{x}_\nu)\right] \tag{108}$$

$$\mathrm{Var}(I) = \frac{1}{n^4} \mathbb{E}\left[\left(\sum_\mu (\mathbf{u} \cdot \mathbf{x}_\mu) \operatorname{erf}(\mathbf{w} \cdot \mathbf{x}_\mu)\right)^4\right] - \bar{I}^2.$$

To estimate the scale of these quantities, we focus simplicity on the regime where the error function is linear. This can be generalized by taking into account perturbative corrections, but these do not change the scale. Following this approximation and taking into account centered Gaussian i.i.d. measure with variance 1 for, $d\mu_x$ one finds,

$$\bar{I} = (\mathbf{u} \cdot \mathbf{w})^2 + \frac{1}{n}\left[(\mathbf{u} \cdot \mathbf{w})^2 + \|\mathbf{u}\|^2 \|\mathbf{w}\|^2\right] \tag{109}$$

$$\mathrm{Var}(I) = \frac{3}{n^2} \|\mathbf{u}\|^4 \|\mathbf{w}\|^4 + O((n^2 S)^{-1})$$

Since $\mathbf{u}$ and $\mathbf{w}$ are high- dimensional vectors where $\mathbf{w} \sim \mathcal{N}(0, I_S \sigma^2/S)$ and $\mathbf{u}$ can be taken to be fixed with $O(1)$ norm in our setting. Therefore, the norm of $\mathbf{w}$ concentrates on its average value, σ_{w}^2, which is of order one, with fluctuation of order $S^{-1/2}$. Hence, the variance fluctuation are of order $1/n^2$ and mean fluctuation are of order $\max(1/S, 1/n)$. Thus, $n \gg S$ is required for replacing the summation by an integral for $N = 1$ (the fully connected case). Similar analysis can be done for general N leading to $Var(I) = O(1/(Nn^2))$, and $\bar{I} = O(1/(SN))$ which then requires $n \gg \sqrt{N}S$, where we assumed, without loss of generality, that $\mathbf{u}_j = a_j^* \mathbf{w}^*$ and $\|\mathbf{a}^*\|^2 = 1$, as in the previous section. For our CNN model $N = 20, S = 64$ and $n = 800, 1600$ hence this approximation is reasonable.

Let us consider the implications this has on the fully connected case. In the regime where $n \gg S$, the behavior of the MSE will change drastically compared to $N \gg 1$. Indeed, as our previous results show, for our CNN experiments,

the train MSE (over σ^4) reaches 0.4^2 of the corresponding at $n = 1600 \gg S$, and for the GP this quantity is order 1. Thus, while being small, this MSE is far from negligible. Specifically, in our CNN experiments, the emergent scale ($\alpha^2 n^2/(CNS)$) is order 1.

In contrast, at $N = 1$, much like in experiments [10], the self-consistent equation predicts a negligible GP-DNN performance gap down to C or order 1. Moreover, for $n \gg S$ the GP (which has a uniform prior over all the S possible linear functions) actually performs very well. Specifically, the EK approximation yields a train MSE (over σ^4) of order 10^{-5}. The emergent scale ($\alpha^2 n^2/(CS)$) is of the same order at $1/C$.

Several conclusions could be drawn here: (i) Taking $N = 1$ in our experiments, there is no separation of scales between the emergent scale and $1/C$. (ii) In a related manner, no appreciable label/target-aware feature learning will take place down to the scale where our inter-layer mean-field breaks down ($C \sim 1$).

4 Supplementary Note - Validity of the Variational Gaussian Approximation

Here, we provide some analytical support to the validity of the Gaussian variational approximation, used to obtain the equation of state. We present the variational treatment from a perturbation theory approach, as a partial summation of a subset of all perturbative corrections. We identify a qualitative difference between this subset and other perturbative corrections that we neglect. We apply our analysis to one of the typical hidden layers in the mean-field limit. This is easily generalized to all layers due to the recursive structure of the problem.

In Eq. (27), we introduce the mean-field probability distribution:

$$\pi^{(l)} \propto \prod_c e^{-\mathcal{S}^{(l)}_{c,\mathrm{MF}}} \tag{110}$$

where here we separate the action into two parts $\mathcal{S}^{(l)}_{c,\mathrm{MF}} = \mathcal{S}^{(l)}_{0c,\mathrm{MF}} + \frac{1}{C}\Delta\mathcal{S}^{(l)}_{c,\mathrm{MF}}$. Here we denote by $\mathcal{S}^{(l)}_{0c,\mathrm{MF}} = \frac{1}{2}\left(\mathbf{h}^{(l)}_c\right)^{\mathsf{T}}\left[Q^{(l)}\right]^{-1}\mathbf{h}^{(l)}_c$, the action of the free theory and by $\Delta\mathcal{S}^{(l)}_{c,\mathrm{MF}} = -\frac{1}{2}\frac{\sigma_l^2}{N_{l-1}}\sum_{\mu\nu} A^{(l+1)}_{\mu\nu}\phi\left(h^{(l)}_{c\mu}\right)\phi\left(h^{(l)}_{c\nu}\right)$ where $A^{(l+1)} = \langle \mathbf{m}^{(l+1)}\left(\mathbf{m}^{(l+1)}\right)^{\mathsf{T}}\rangle_{\mathrm{MF}}$ the action coming from the interaction between layers. σ_l^2 is the variance of the weights of the lth layer. Since all the channels are independent, in the following, we drop the channel index (c). For compactness of notation, we also drop the index of the layer (l) and the indices μ and ν represents the multi-index over the strides and the training data samples. In the variational Gaussian approximation, we replace this probability with a Gaussian one with covariance matrix K which minimizes the KL divergence between the

two distributions. The KL divergence between the two distributions is as follows:

$$D_{KL}(\mathcal{N}(0,K)||\pi) = -\langle\frac{1}{2}\mathbf{h}^{\mathsf{T}}Q^{-1}\mathbf{h}\rangle_K + \langle\frac{1}{2}\frac{\sigma_l^2}{CN}\sum_{\mu\nu}A_{\mu\nu}\left[\phi\left(h_{c\mu}\right)\phi\left(h_{c\nu}\right)\right]\rangle_K + \frac{1}{2}\log\det(K) + C. \quad (111)$$

where $\langle ... \rangle_K$ is averaging with respect to the Gaussian measures induced by the kernel K. For the layer below, This yields the following self-consistent equation for the post-kernel, Q and the pre-kernel, K:

$$Q^{-1} - \frac{1}{C}\sum_{\mu\nu}A_{\mu\nu}\left[\partial_K Q\right]_{\mu\nu} = K^{-1}. \quad (112)$$

We now show in what sense this approximation is valid, i.e. when can we approximate the mean-field distribution by a Gaussian distribution. We start by calculating the interacting Green function of the process (second moment of the process).

$$\langle h_\mu h_\nu\rangle = \langle h_\mu h_\nu\rangle_0 + \sum_{j=1}^{\infty}\frac{1}{C^j j!}\langle h_\mu h_\nu\left(\Delta\mathcal{S}\right)^j\rangle_0, \quad (113)$$

where $\langle\ldots\rangle$ is the connected expectation with respect to $\mathcal{S}_{\mathrm{MF}}$ and $\langle\ldots\rangle_0$ is the connected expectation with respect to $\mathcal{S}_{0,\mathrm{MF}}$. Here, connected mean cumulant moments w.r.t the variables hh and $\Delta\mathcal{S}_{\mathrm{MF}}$. We perform our perturbation analysis, for simplicity, for the monomial activation function $\phi(x) = x^k$, with k finite, as a characteristic example. This can be generalized to other smooth activation functions. The perturbative correction term, to the free moment, $\langle h_\mu h_\nu\left(\Delta S_{\mathrm{MF}}\right)^j\rangle_0$ is as follows:

$$\langle h_\mu h_\nu\left(\sum_{\alpha_1\beta_1}A_{\alpha_1\beta_1}h_{\alpha_1}^k h_{\beta_1}^k\right)^j\rangle_0 = \sum_{\alpha_1\beta_1\ldots\alpha_j\beta_j}A_{\alpha_1\beta_1}\ldots A_{\alpha_j\beta_j}\langle h_\mu h_\nu h_{\alpha_1}^k h_{\beta_1}^k\ldots h_{\alpha_j}^k h_{\beta_j}^k\rangle_0, \quad (114)$$

where $\langle h_\mu h_\nu\rangle_0 = Q_{\mu\nu}$. In order to evaluate the above expression, we need to introduce some additional assumption on the structure of the matrix Q. Based on the numerical simulation and since this matrix represents the correlation between samples in high dimension, we consider the following estimate on the elements of the matrix Q, off-diagonal elements, $Q_{\mu\nu} = O(m^{-1/2})$, and on-diagonal elements, $Q_{\mu\mu} = O(1)$, for $Q \in \mathbb{R}^{m\times m}$, where for FCN $m = n$ number of samples for the 3-layer CNN $m = nS_1$ where S_1 is the size of the stride. Under this structure of the matrix Q, the leading terms in our perturbation expansions for large m are

of the following forms (connected diagrams only), other terms will be of higher powers of $m^{-1/2}$,

$$\begin{aligned}
&\sum_{\alpha_1\beta_1\ldots\alpha_j\beta_j} k^{2j} A_{\alpha_1\beta_1}\ldots A_{\alpha_j\beta_j}\langle h_\mu h_{\alpha_1}\langle h_{\alpha_1}^{k-1}h_{\beta_1}^{k-1}\rangle_0 h_{\beta_1}\ldots h_{\alpha_j}\langle h_{\alpha_j}^{k-1}h_{\beta_j}^{k-1}\rangle_0 h_{\beta_j}h_\nu\rangle_0 \\
&= \sum_{\alpha_1\beta_1\ldots\alpha_j\beta_j} k^{2j} j! A_{\alpha_1\beta_1}\ldots A_{\alpha_j\beta_j}\langle h_\mu h_{\alpha_1}\rangle_0\langle h_{\alpha_1}^{k-1}h_{\beta_1}^{k-1}\rangle_0\langle h_{\beta_1}h_{\alpha_2}\rangle_0\ldots\langle h_{\beta_{j-1}}h_{\alpha_j}\rangle_0\langle h_{\alpha_j}^{k-1}h_{\beta_j}^{k-1}\rangle_0\langle h_{\beta_j}h_\nu\rangle_0 \\
&= \sum_{\alpha_1\beta_1\ldots\alpha_j\beta_j} k^{2j} j! 2^j Q_{\mu\alpha_1}\langle h_{\alpha_1}^{k-1}h_{\beta_1}^{k-1}\rangle_0 A_{\alpha_1\beta_1}Q_{\beta_1\alpha_2}\ldots Q_{\beta_{j-1}\alpha_j}\langle h_{\alpha_j}^{k-1}h_{\beta_j}^{k-1}\rangle_0 A_{\alpha_j\beta_j}Q_{\beta_j\nu} \\
&= \sum_{\beta_1\ldots\beta_j} k^{2j} j!\,[QV(k)]_{\mu\beta_1}[QV(k)]_{\beta_1\beta_2}\ldots[QV(k)]_{\beta_{j-1}\beta_j}Q_{\beta_j\nu} \\
&= \sum_\beta k^{2j} j!\left[(QV(k))^j\right]_{\mu\beta}Q_{\beta\nu}, \quad (115)
\end{aligned}$$

where k^{2j} is due to the choice of h_α from each monomial activation function, the $j!$ is due to the arrangement in pairs of the remaining fields. We denote by $V(k)_{\alpha_1\beta_1} = \langle h_{\alpha_1}^{k-1}h_{\beta_1}^{k-1}\rangle_0 A_{\alpha_1\beta_1}$. Plugging back in Eq. (113) leads to the following self-consistent equation:

$$\begin{aligned}
K_{\mu\nu} &= Q_{\mu\nu} + \sum_{j=1}^{\infty}\sum_\beta\left[\left(\frac{2k^2}{C}QV(k)\right)^j\right]_{\mu\beta}Q_{\beta\nu} + O(m^{-1/2}) \\
&= \sum_\beta\left[\left(I_m - \frac{2k^2}{C}QV(k)\right)^{-1}\right]_{\mu\beta}Q_{\beta\nu} + O(m^{-1/2}) \quad (116)
\end{aligned}$$

In the first transition, we sum over the geometric series in the regime, where $\|QV(k)\|_{\text{op}} < C/2k^2$, which is valid for large enough C, and m. We now invert the equation in order to compare to our mean-field equation (Eq. (112)):

$$K^{-1} = Q^{-1} - \frac{2k^2}{C}QV(k) + O(\left(C\sqrt{m}\right)^{-1})$$

Plugging the definition of the matrix V, we have that,

$$\begin{aligned}
\left[K^{-1}\right]_{\mu\nu} &= \left[Q^{-1}\right]_{\mu\nu} - \frac{k^2}{C}\sum_\rho Q_{\mu\rho}\langle\, h_\rho^{k-1}h_\nu^{k-1}\rangle_0 A_{\rho\nu} + O(\left(C\sqrt{m}\right)^{-1}) \\
&= \left[Q^{-1}\right]_{\mu\nu} - \frac{1}{C}\sum_\rho \partial_{Q_{\mu\rho}}\langle\, h_\rho^k h_\nu^k\rangle_0 A_{\rho\nu} + O(\left(C\sqrt{m}\right)^{-1})
\end{aligned}$$

The second transition is using Gaussian integration by parts. The resulting equation is very similar to the mean-field equation we find. The difference is that here instead of derivative by K, the pre-kernel, we have a derivative of the post-kernel. In addition, the expectation is also with respect to the free theory. Indeed, the full variational treatment is self-consistent or, equivalently

stated, it takes into account a larger set of diagrams (terms in perturbation theory) which amount to renormalizing the 2-point function from Q to K. We argue however that doing so only improves the overall accuracy. Indeed, the expansion of Eq. (115), is the same as one would get from a Gaussian action consisting of $\mathcal{S}_{0,\mathrm{MF}}$ plus a quadratic term of the form $h_{\alpha_j} h_{\beta_j} k \langle h^{k-1}_{\alpha_j} h^{k-1}_{\beta_j} \rangle A_{\alpha_j \beta_j}$. The variational Gaussian approximation essentially finds the closest Gaussian distribution. Therefore, it can only improve upon this simpler approximation we took here.

5 Supplementary Note - Variational Gaussian Approximation for ReLU Activation

Here, we extend the previous VGA treatment, which assumed centered distributions, to non-centered ones. Indeed, for antisymmetric activation functions, the pre-activations appear schematically as $\mathbf{h}^{(l+1)} = \mathbf{v}\phi(\mathbf{h}^{(l)})$, thus $\mathbf{v}\phi(\mathbf{h}^{(l)}) = -\mathbf{v}\phi(-\mathbf{h}^{(l)})$. Since $\mathbf{v}$ appears only in quadratic order in the action, we find that $\mathbf{h}^{(l)}$ is as likely as $-\mathbf{h}^{(l)}$ and its ensemble average is strictly zero. However, for $\phi = \mathrm{ReLU}$, it is not the case. This requires us to extend the previous treatment by including extra variational parameters for the mean.

Concretely, let us focus on the VGA for the input layer of the 3 layer CNN considered in the main text. Our VGA for the probability is now defined by the variance of the Gaussian in weight space, $\Sigma_{ss'}$ as well as the mean χ_s. Repeating the previous analysis one obtains

$$
\begin{aligned}
D(\mathcal{N}(\boldsymbol{\chi}, \Sigma) | \pi(\mathbf{w}_c | X_n; \langle \mathbf{m}\mathbf{m}^{\mathsf{T}} \rangle_{\mathrm{MF}})) &= -\frac{S_0}{2\sigma_{\mathrm{w}}^2} \left[\mathrm{Tr}\,(\Sigma) + \|\boldsymbol{\chi}\|^2 \right] \\
&- \frac{C_2}{2C_1} \sum_{\mu\nu j_1 j_2} \left(\langle \mathbf{m}\mathbf{m}^{\mathsf{T}} \rangle_{\mathrm{MF}} \right)_{\mu\nu j_1 j_2} \left[Q^{(2)} \right]_{\mu\nu j_1 j_2} + \frac{1}{2} \log\det(\Sigma) + \mathrm{Const},
\end{aligned}
$$

where $\mathbf{w}_c \in R^{S_0}$ are the weights of channel, c and $Q^{(2)}_{\mu j_1 \nu j_2}$ is now defined by

$$
Q^{(2)}_{\mu j_1 \nu j_2} = \left\langle \frac{\sigma_{\mathrm{v}}^2}{S_1 C_1} \sum_{ic}^{S_1} \phi\left(\mathbf{w}_c \cdot \mathbf{x}_{\mu, i + j_1 S_1} \right) \phi\left(\mathbf{w}_c \cdot \mathbf{x}_{\nu, i + j_2 S_1} \right) \right\rangle_{\Sigma, \boldsymbol{\chi}}, \tag{117}
$$

which one can reduce to a one-dimensional integral following Ref. [11]. Obtaining an explicit expression, or potentially a perturbative expansion in $\boldsymbol{\chi} \cdot \mathbf{x}_{\mu,i}$, is left for future work.

Taking the derivative of the KL-divergence with respect to Σ and equating it to zero, one obtains

$$
\Sigma^{-1} = \frac{S_0}{\sigma_{\mathrm{w}}^2} I_{S_0} + \frac{C_2}{C_1} \sum_{\mu\nu j_1 j_2} \left(\langle \mathbf{m}\mathbf{m}^{\mathsf{T}} \rangle_{\mathrm{MF}} \right)_{\mu j_1 \nu j_2} \left[\partial_\Sigma Q^{(2)} \right]_{\mu j_1, \nu j_2}. \tag{118}
$$

Differentiating w.r.t. $\boldsymbol{\chi}$ yields the additional equation

$$\chi_{\mu j} = -\frac{C_2 \sigma_{\mathrm{w}}^2}{2 C_1 S_0} \sum_{\mu j_1, \nu j_2} \left(\langle \mathbf{m} \mathbf{m}^{\mathsf{T}} \rangle_{\mathrm{MF}} \right)_{\mu j_1, \nu j_2} \left[\partial_{\chi_{\mu j}} Q^{(2)} \right]_{\mu j_1, \nu j_2} \tag{119}$$

This leaves the quantity $\langle \mathbf{m} \mathbf{m}^{\mathsf{T}} \rangle_{\mathrm{MF}}$. Repeating the derivation in subsection 1.4.2 noticing that now $\langle \mathbf{h}^{(2)} \rangle_{\mathrm{MF}}$ is non-zero, we obtain

$$\langle \mathbf{m} \mathbf{m}^{\mathsf{T}} \rangle_{\mathrm{MF}} = Q^{(2)} - [Q^{(2)}]^{-1} \left[K^{(2)} + \boldsymbol{\chi}^{(2)} (\boldsymbol{\chi}^{(2)})^{\mathsf{T}} \right] [Q^{(2)}]^{-1} \tag{120}$$

where $\chi_{\mu j}^{(2)}$ is the average of $h_{\mu j c'}^{(2)}$ (for any c').

6 Supplementary Note - Further details on the numerical experiments

6.1 FCN experiments

Here we report on several additional numerical results for FCNs. In particular, we provide details about the full spectrum of Σ, the equilibration process, and the Gaussianity measures. We also report on some numerical experiments with FCN in the standard scaling.

We conducted further experiments with the above FCNs ($d = 64, n = 1024, \sigma^2 = 0.001$, teacher-student with $N_l = 1$ for the teacher) however with standard scaling ($\sigma_{\mathrm{a}}^2 = 2$, regardless of N_l) rather than "MF" scaling ($\sigma_{\mathrm{a}}^2 = 2/N_2$). Here, we expect feature learning to diminish [12]. Considering the numerical solution of our EoS, we found that they essentially remain close to the GP limit for $N_1 = N_2 = 64$. Specifically, at $N_1 = N_2 = 64$ leading Σ eigenvalue came out 0.03257, whereas in the GP limit we obtain $2/d = 0.03125$. This is consistent with the fact that the emergent scale here (Supplementary Figure 1. panel (b) main text) is of the order of $1/N_2$. Supplementary Figure 1 and Supplementary Figure 2 presents the results for different width N_l.

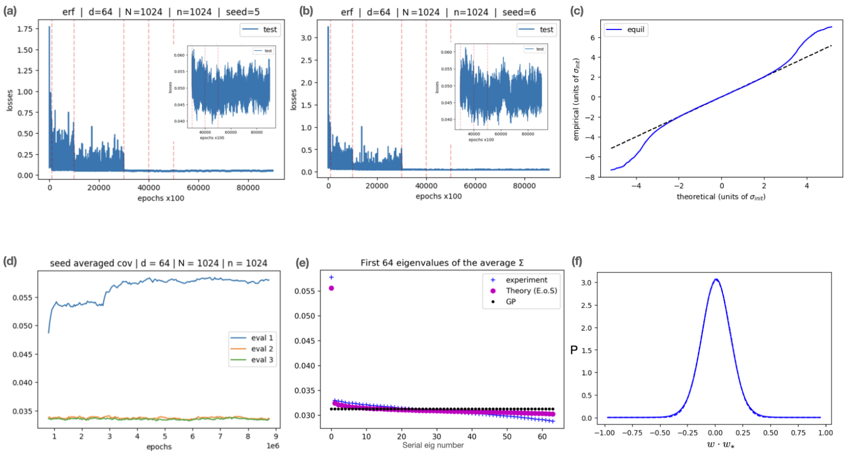

Supplementary Figure 1: **FCN "MF". Theory versus Experiment.** $N_1 = N_2 = 1024, \sigma^2 = 0.001$, 26 training seeds. Further experimental data on $d = 64, n = 1024$ FCN with MF scaling is reported in Fig. 1. panel (b) of the main text. Panels (**a**,**b**) show the test loss as a function of the number of epochs, with insets focusing on later times. Panels (**c**,**f**) study the distribution $P(\mathbf{w} \cdot \mathbf{w}^*)$ as a QQ-plot against a Gaussian and as a histogram with the least square fit to a Gaussian (the latter in dashed lines). Panel (**d**) Shows the equilibration Σ averaged over the different training-seed, via its 3 leading eigenvalue. Panel (**e**) shows the empirical, GP, and theoretical eigenvalues of the average Σ. As before, by GP here we mean taking $N_1, N_2 \to \infty$ at fixed σ_{a}^2.

6.2 2-layer CNN experiment

Supplementary Figure 3 shows the top Σ eigenvalues normalized by σ_{w}^2/d, this is a complementary figure to Figure 1(b) introduced in the main text. Clearly, as the width increase, the eigenvalues of Σ are getting closer to the GP kennel eigenvalues.

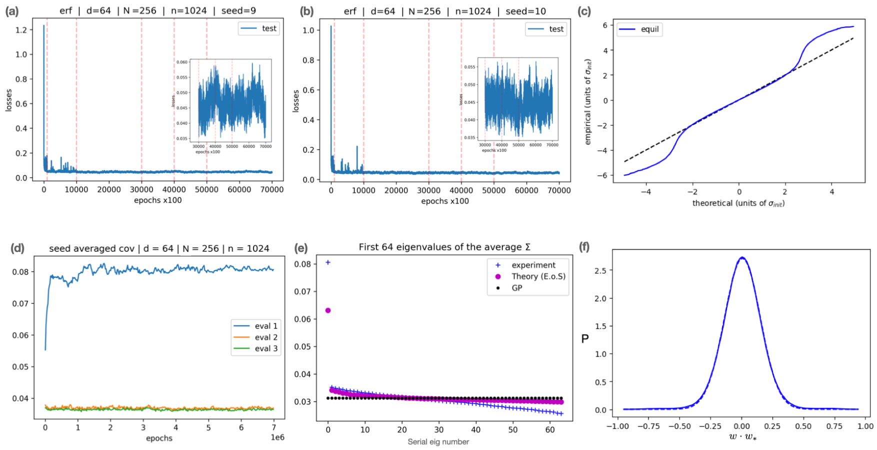

Supplementary Figure 2: **FCN "MF". Theory versus Experiment.** $N_1 = N_2 = 256$. 27 training seeds. $\sigma^2 = 0.001$. See the previous caption for the panel description.

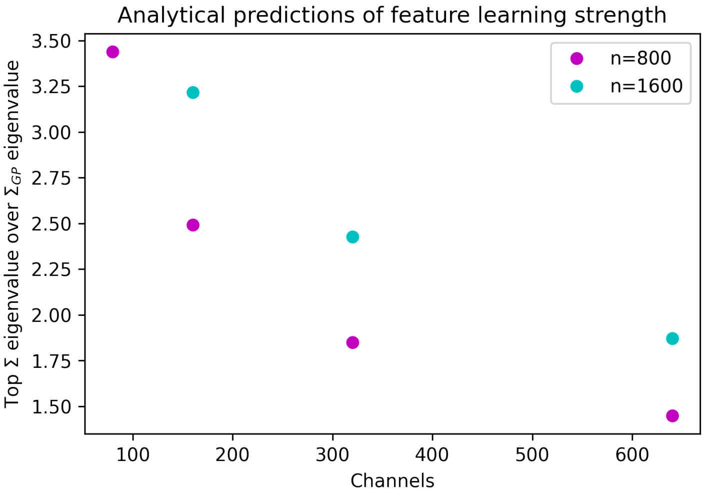

Supplementary Figure 3: **2-Layer CNN, Sigma eigenvalues.** Top Σ eigenvalue according to theory over σ_{w}^2/d which is the (degenerate) Σ eigenvalue in the GP limit, for the experiment shown for the 2-layer CNN in the main text.

6.3 Myrtle-5 CNN on subsets of CIFAR-10 experiment

Here, we report the statistics of the pre-activations in Fourier space similar to Figure 6 in the main text, but now for all layers and projected on the 1st, 3rd and 10th eigenvectors of the Fourier space covariance matrix. Let us begin by giving some more details on the procedure for deriving these quantities. The pre-activations at some layer is of the form $h_{\mu,c,x,y}$ where μ is a data point index, c is a channel index, and x, y are pixel coordinates. We choose some wavenumber k and transform these to Fourier space to yield $h^k_{\mu,c}$ which summarizes contributions from all pixels. We then compute the covariance matrix of these $h^k_{\mu,c}$ (an $n \times n$ matrix), averaging across channels and seeds. Finally, we project $h^k_{\mu,c}$ on some eigenvector of the covariance matrix, and these are the quantities whose statistics we report in Supplementary Figures 4, 5.

There are several empirical observations to be made here:

1. Gaussianity generally increases as we go deeper into the network from the input to the output.
2. Gaussianity generally increases as we project on higher index eigenvectors (going from left to right across the columns).
3. Deviations from Gaussianity can appear in several ways: e.g. as multi-modality (e.g. top left panel), or as excessive kurtosis (e.g. 2nd-row left column).

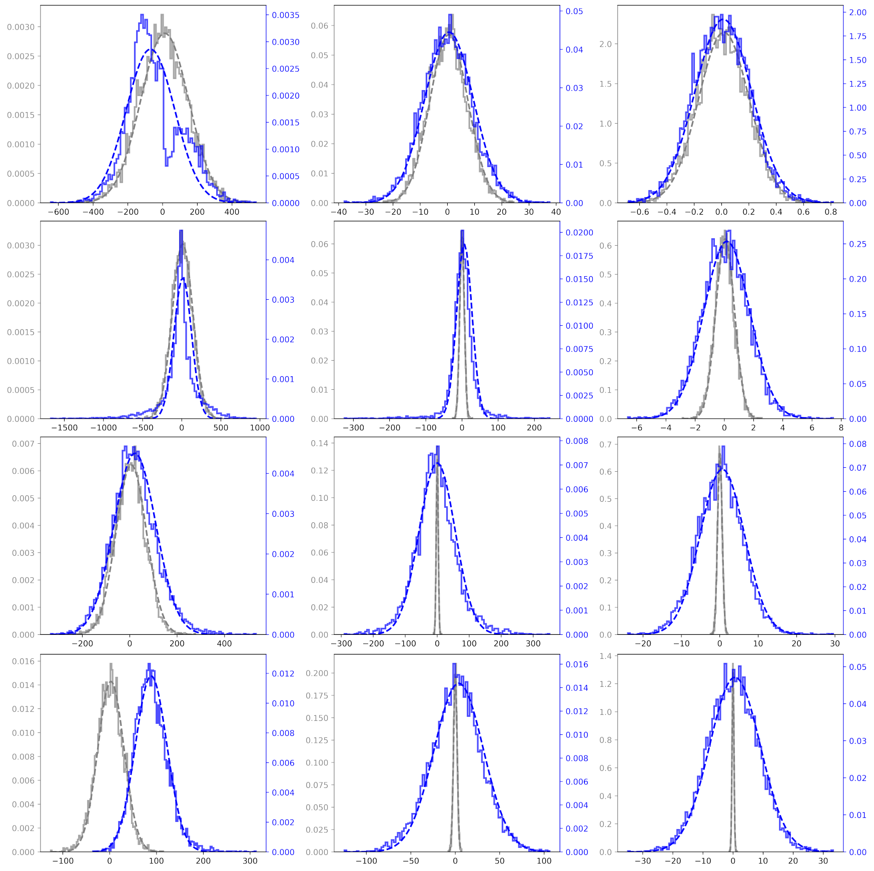

Supplementary Figure 4: **Pre-activation statistics of Myrtle-5 CNN:** Grey and blue lines denote the statistics of untrained nets with random initialization and nets at equilibrium, respectively. Dashed lines are Gaussian fits with non-linear least squares to the empirical distributions. Each row corresponds to a specific layer in the network, and each column corresponds to a projection to a different eigenvector of the covariance matrix in Fourier space: the 1st, 3rd and 10th eigenvectors, respectively.

We further report the statistics of the inter-layer and inter-channel correlations in Table 1 and Table 2, respectively. We report the value of the estimator of correlations together with the error due to finite samples (i.e. mean $\pm$ std of the estimator). We see that the correlations across different layers, as well as across different channels within the same layer are mostly on the order of 10^{-3} (apart from the inter-channel correlations in layer 4 which are slightly larger). The larger error we see for the estimator of the inter-channel correlations is a result of having a smaller sample size since for the inter-layer correlations the number

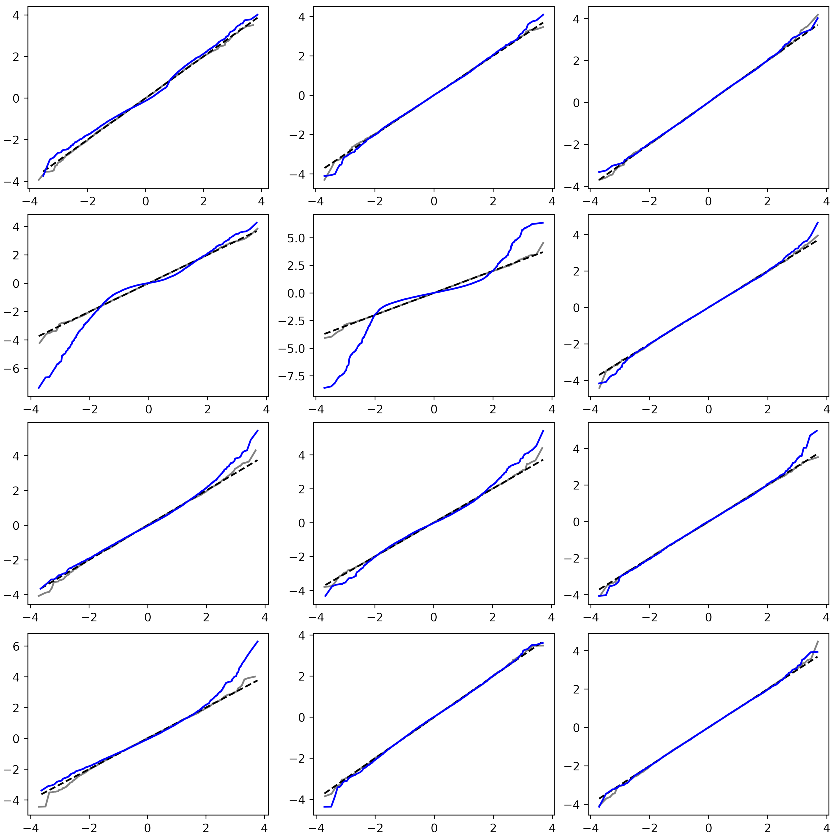

Supplementary Figure 5: **Pre-activation statistics of Myrtle-5 CNN - QQ plots:** Here we show the QQ plots corresponding to the same data appearing in the previous figure. The x-axis corresponds to the best fit Gaussian distribution, and the y-axis corresponds to the empirical distribution (both in units of the std at initialization). The black dashed line corresponds to the identity line, and the closer the curve is to it, the better the fit to a Gaussian. Grey and blue lines denote the statistics of untrained nets with random initialization and trained nets at equilibrium, respectively. Dashed lines are Gaussian fits with non-linear least squares to the empirical distributions. Each row corresponds to a specific layer in the network, and each column corresponds to a projection to a different eigenvector of the covariance matrix in Fourier space: the 1st, 3rd and 10th eigenvectors, respectively.

of samples is $n_{\text{seeds}} \cdot C$ while for the inter-channel correlations the number of samples is only n_{seeds} (where the number of seeds used for this experiment is $n_{\text{seeds}} = 25$, and recall that the number of channels here is $C = 256$). These

empirical findings justify our approximations which neglect inter-channel and inter-layer correlations.

	layer 2	layer 3	layer 4
layer 1	-9.58e-03 ± 5.02e-03	-8.73e-03 ± 6.77e-03	4.51e-03 ± 8.53e-03
layer 2		-1.50e-02 ± 8.94e-03	-6.85e-03 ± 6.82e-03
layer 3			-9.27e-05 ± 1.02e-02

Supplementary Table 1: Inter-layer Pearson correlations

layer 1	layer 2	layer 3	layer 4
-0.0041 ± 0.2057	0.0079 ± 0.2057	-0.0031 ± 0.2051	0.0185 ± 0.2045

Supplementary Table 2: Inter-channel Pearson correlations

Supplementary References

[1] Williams, C. K. Computing with infinite networks. In *Advances in neural information processing systems*, 295–301 (1997). 1.3.2

[2] Bordelon, B., Canatar, A. & Pehlevan, C. Spectrum dependent learning curves in kernel regression and wide neural networks (2020). `2002.02561`. 1.6, 3

[3] Yu, J. & Spiliopoulos, K. Normalization effects on shallow neural networks and related asymptotic expansions. *arXiv e-prints* arXiv:2011.10487 (2020). `2011.10487`. 1.6

[4] Rasmussen, C. E. & Williams, C. K. I. *Gaussian Processes for Machine Learning (Adaptive Computation and Machine Learning)* (The MIT Press, 2005). 3

[5] Cohen, O., Malka, O. & Ringel, Z. Learning Curves for Deep Neural Networks: A Gaussian Field Theory Perspective. *arXiv e-prints* arXiv:1906.05301 (2019). `1906.05301`. 3, 3

[6] Naveh, G., Ben David, O., Sompolinsky, H. & Ringel, Z. Predicting the outputs of finite deep neural networks trained with noisy gradients. *Physical Review E* **104** (2021). URL `http://dx.doi.org/10.1103/PhysRevE.104.064301`. 3

[7] Bordelon, B., Canatar, A. & Pehlevan, C. Spectrum dependent learning curves in kernel regression and wide neural networks (2021). `2002.02561`. 3

[8] Simon, J. B., Dickens, M. & DeWeese, M. R. A Theory of the Inductive Bias and Generalization of Kernel Regression and Wide Neural Networks. *arXiv e-prints* arXiv:2110.03922 (2021). `2110.03922`. 3

[9] Naveh, G. & Ringel, Z. A self consistent theory of gaussian processes captures feature learning effects in finite cnns. *Advances in Neural Information Processing Systems* **34** (2021). 3

[10] Lee, J. *et al.* Deep neural networks as gaussian processes. In *International Conference on Learning Representations* (2018). URL `https://openreview.net/forum?id=B1EA-M-0Z`. 3.1

[11] Cho, Y. & Saul, L. K. Kernel Methods for Deep Learning. *NIPS* 1–9 (2009). 5

[12] Yang, G. Scaling limits of wide neural networks with weight sharing: Gaussian process behavior, gradient independence, and neural tangent kernel derivation. *arXiv preprint arXiv:1902.04760* (2019). 6.1
